# LncRNA PKMYT1AR promotes cancer stem cell maintenance in non-small cell lung cancer via activating Wnt signaling pathway

**DOI:** 10.1186/s12943-021-01469-6

**Published:** 2021-12-02

**Authors:** Yaomei He, Xiulin Jiang, Lincan Duan, Qiuxia Xiong, Yixiao Yuan, Peishen Liu, Liping Jiang, Qiushuo Shen, Song Zhao, Cuiping Yang, Yongbin Chen

**Affiliations:** 1 Key Laboratory of Animal Models and Human Disease Mechanisms of Chinese Academy of Sciences & Yunnan Province, Kunming Institute of Zoology, Kunming, 650223 Yunnan China; 2 Kunming College of Life Science, University of Chinese Academy of Sciences, Beijing, 100049 China; 3 Department of Thoracic Surgery, the Third Affiliated Hospital of Kunming Medical University, Kunming, 650118 Yunnan China; 4 Department of Clinical Laboratory, the First Affiliated Hospital of Kunming Medical University, Kunming, 650032 China; 5 Department of Thoracic Surgery, the First Affiliated Hospital of Zhengzhou University, Zhengzhou, 450052 China; 6 Center for Excellence in Animal Evolution and Genetics, Chinese Academy of Sciences, Kunming, 650223 Yunnan China

**Keywords:** PKMYT1AR, miR-485-5p, PKMYT1, Non-small cell lung cancer, Cancer stem cells (CSCs)

## Abstract

**Background:**

Non-small cell lung cancer (NSCLC) is the most common type of human lung cancers, which has diverse pathological features. Although many signaling pathways and therapeutic targets have been defined to play important roles in NSCLC, limiting efficacies have been achieved.

**Methods:**

Bioinformatics methods were used to identify differential long non-coding RNA expression in NSCLC. Real-time RT-PCR experiments were used to examine the expression pattern of lncRNA PKMYT1AR, miR-485-5p. Both in vitro and in vivo functional assays were performed to investigate the functional role of PKMYT1AR/miR-485-5p/PKMYT1 axis on regulating cell proliferation, migration and tumor growth. Dual luciferase reporter assay, fluorescent in situ hybridization (FISH), immunoblot, co-immunoprecipitation experiments were used to verify the molecular mechanism.

**Result:**

Here, we identify a human-specific long non-coding RNA (lncRNA, ENST00000595422), termed PKMYT1AR (PKMYT1 associated lncRNA), that is induced in NSCLC by Yin Yang 1 (YY1) factor, especially in cancerous cell lines (H358, H1975, H1299, H1650, A549 and SPC-A1) compared to that in normal human bronchial epithelium cell line (BEAS-2B). We show that PKMYT1AR high expression correlates with worse clinical outcome, and knockdown of PKMYT1AR inhibits tumor cell proliferation, migration and xenograft tumor formation abilities. Bioinformatic analysis and a luciferase assay demonstrate that PKMYT1AR directly interacts with miR-485-5p to attenuate the inhibitory role on its downstream oncogenic factor PKMYT1 (the protein kinase, membrane-associated tyrosine/threonine 1) in NSCLC. Furthermore, we uncover that miR-485-5p is downregulated in both cancerous cell lines and peripheral blood serum isolated from NSCLC patients compared to reciprocal control groups. Consistently, forced expression of miR-485-5p inhibits the proliferation and migration abilities of tumor cells. Moreover, we provide evidence showing that PKMYT1AR targeting antisense oligonucleotide (ASO) dramatically inhibit tumor growth in vivo. Mechanistic study shows that PKMYT1AR/ miR-485-5p /PKMYT1 axis promotes cancer stem cells (CSCs) maintenance in NSCLC via inhibiting β-TrCP1 mediated ubiquitin degradation of β-catenin proteins, which in turn causes enhanced tumorigenesis.

**Conclusions:**

Our findings reveal the critical role of PKMYT1AR/miR-485-5p /PKMYT1 axis during NSCLC progression, which could be used as novel therapeutic targets in the future.

**Supplementary Information:**

The online version contains supplementary material available at 10.1186/s12943-021-01469-6.

## Background

Lung cancer is a fatal malignant tumor originated from bronchial mucosa or glands, which could be divided into small cell lung cancer (SCLC) and non-small cell lung cancer (NSCLC). SCLC and NSCLC account for about 20 and 80% of lung cancer cases respectively, while NSCLC could be further subdivided into adenocarcinoma (LUAD), squamous cell carcinoma (LUSC) and large cell lung cancer [1]. Therapeutic option for the early stage of lung cancer was a comprehensive treatment based on the surgery, while the multidisciplinary treatment plays an important role in advanced non-small cell lung cancer, and radiotherapy, chemotherapy, immunotherapy, targeted treatment or combined treatment are the main treatment strategies [2]. The incidence of NSCLC is relatively high, with the overall 5-year survival lower than 17%, although the diagnosis and treatment strategies for NSCLC have improved greatly in recent years [3]. Therefore, it is still imperative to investigate the molecular mechanisms regulating lung cancer progression, and find new therapeutic targets improving the clinical outcome.

Long non-coding RNAs (lncRNAs) are longer than 200 nucleotides barely harboring protein-coding potential, which have been uncovered to play pivotal roles in lung cancer, although their functional importance and molecular mechanisms await further investigation [4]. LncRNAs regulate tumor progression by diverse mechanisms, while the functional role of lncRNAs as competing endogenous RNAs (ceRNAs) has received great attention as a “microRNA (miRNA) sponge” to eliminate miRNA-mediated inhibition of targeted gene [5]. MicroRNAs are a class of short (with an average of 18-25 nucleotides) endogenously initiated non-coding RNAs that regulate gene expression by binding to targeted mRNA 3’-untranslated region (3’-UTR) to inhibit the stability of mRNA and translational efficiency of targeted mRNAs [6]. As a result, miRNAs could decrease the expression of tumor suppressors, leading to increased expression of oncogenes, during the occurrence or progression of lung cancer [7]. Based on the fact that non-coding RNAs are involved in modulating chemo- or radio-therapy sensitivity, as well as targeted drug therapy in lung cancer, nucleic acid-based strategies either by controlling the expression levels of lncRNAs or modifying their native structures prevail in targeting RNA have been developed [8]. Among them, RNA interference (RNAi) based techniques and antisense oligonucleotide (ASO) were widely applied [8].

An increasing number of recent studies have demonstrated that cancer stem cells (CSCs) have the stem cell biological characteristics, such as the ability of self-renewal and differentiation, and are important for tumor metastasis and drug resistance, with their potential clinical significance and molecular mechanism unsolved. In addition, many documented findings demonstrate that lncRNAs or miRNAs play essential roles in CSCs during tumor progression [9, 10]. Mechanistic studies have revealed that multiple signaling pathways (eg. Wnt/β-catenin, Hedgehog and Notch) essential for normal stem cell self-renewal, are critical for regulating the stemness maintenance of CSCs, and various CSCs marker genes have been defined in lung cancer, including CD133, CD44, ALCAM and CD90 [11].

PKMYT1 (the protein kinase, membrane-associated tyrosine/threonine 1), one member of WEE family kinases, was discovered to inhibit Cdk1 phosphorylation during cell cycle transition [12]. Since then, multiple studies have uncovered the oncogenic role of PKMYT1 in different types of human cancers [13–15]. However, the underlying mechanism by which how PKMYT1 is upregualted and its specific downstream targeted gene in NSCLC remains unclear. In this study, we identified a 539-bp lncRNA, termed PKMYT1AR (PKMYT1 associated lncRNA: ENST00000595422), is highly expressed in NSCLC cancerous tissues and cell lines, which predicts poor prognosis. Next, we found that PKMYT1AR promotes the proliferation, migration, stemness maintenance and xenograft tumor formation abilities of NSCLC tumor cells. Furthermore, we show that PKMYT1AR upregulates PKMYT1 expression by sponging miR-485-5p to abrogate its inhibitory role on PKMYT1. Therefore, we decided to validate whether PKMYT1AR, miR-485-5p or/and PKMYT1 could be used as novel therapeutic targets for NSCLC in the future, and decipher how PKMYT1AR/miR-485-5p/PKMYT1 axis regulates NSCLC progression both in vitro and in vivo.

## Materials and methods

### Cell culture

BEAS-2B cell line was purchased from Cell Bank of Kunming Institute of Zoology, and cultured in BEGM media (Lonza, CC-3170). HEK-293T was obtained from ATCC. Lung cancer cell lines, including A549, H1299, H1975, H838, H1650 and SPC-A1, were purchased from Cobioer, China with STR document, and were cultured in RPMI-1640 medium (Corning) supplemented with 10% fetal bovine serum (FBS) and 1% penicillin/streptomycin. HEK-293T cells were cultured in DMEM medium (Corning).

### Constructs, transfection and lenti-viral infection

PKMYT1AR was generated by RT-PCR and sub-cloned into pCDH-MSCV-E2F-eGFP lenti-viral vector or pCDNA3.1 vector with a 3×Flag tag at the C-terminus. PKMYT1 was generated by RT-PCR and sub-cloned into pCDNA3.1 vector with a 6×MYC tag at the C-terminus and 3×HA tag at the N-terminus, respectively. Independent shRNAs targeting PKMYT1AR/PKMYT1 were synthesized and sub-cloned into the lenti-viral vector pLKO.1 (Addgene, Cambridge, USA). The lenti-viruses were generated according to the following protocol. Briefly, PKMYT1AR/PKMYT1 targeting shRNAs, scrambled control shRNA, pCDH-vec or pCDH-PKMYT1AR were transfected into HEK-293T cells with the psPAX2/pMD2.G plasmids (Addgene) mediated by calcium phosphate. After transfection, the cell supernatants were harvested and used to infect A549 or SPC-A1 cells, and the stably lenti-viral infected cells were selected with puromycin. The ASO control or PKMYT1AR-targetign ASOs, miRNA control or miR-485-5p mimics, inhibitor, and miR-485-5p negative control were purchased from RiboBio (China). Cells were transfected with indicated miRNAs or control oligos using Lipofectamine 2000 (Invitrogen), and then collected for various experiments. All the oligo sequences used in this study are listed in Table S2.

### Non-coding RNA examination

Nuclear and cytoplasmic fractions were isolated using the NORGEN kit (Cat. 21000, NORGEN, USA). Briefly, indicated cells were lysed using Cell Fraction Buffer on ice for 10 min. Subsequently, after centrifugation at 5000 g for 5 min at 4 °C, the supernatant or the pellet were collected for further cytoplasmic or nuclear fraction purification, respectively. For RNA fluorescence in situ hybridization (FISH) assay, Cy3-labelled PKMYT1AR probe was designed and synthesized by RiboBio (China), and the FISH kit (RiboBio, Fluorescent In Situ Hybridization Kit, Cat. C10910) was used to detect the non-coding RNA expression pattern following the manufacturer’s instructions. 4,6-diamidino-2-phenylindole (DAPI) was used to indicate nuclear. All images were obtained with an LSM880 NLO (Zeiss) confocal microscope system.

### Cell proliferation, colony formation and tumor sphere formation assays

Cell proliferation, colony formation, tumor sphere formation assay was performed as previously documented [16]. Briefly, for cell proliferation assay, indicated cells were plated into 12-well plates at a density of 1.5×10^4^, the cell numbers were subsequently counted each day using an automatic cell analyzer countstar (Shanghai Ruiyu Biotech Co., China, IC 1000). For colony formation assay, indicated cells were seeded in 6-well plate (China, NEST, Cat. 703001) with 600 cells per well supplemented with 2 mL cell culture medium, and the cell culture medium was changed every 3 days for 2~3 weeks, and then indicated cells were fixed with 4% PFA and stained with 0.5% crystal violet. For tumor sphere formation assay, indicated cells were plated in ultralow-attachment 6-well plate (Corning; Cat. 3471), cultured in serum-free DMEM/F12 supplemented with B27 (Gibco, Cat. 2309544), 20 ng/mL EGF and 20 ng/mL bFGF, and 4 μg/mL heparin. 10-14 days after culture, the spheres were pictured and counted using Nikon inverted microscope (Ti-S).

### Cell migration and cell flow cytometry assays

Cell migration assays was performed as previously documented. Briefly, to produce a wound, the monolayer cells in 6-well plate were scraped in a straight line with pipette tips. Plate was then washed with PBS to remove detached cells. Photographs of the scratch were taken at indicated time points using Nikon inverted microscope (Ti-S). Gap width was calculated using GraphPad Prism software. For trans-well assay, 2.5×10^4^ cells in 100μL serum free medium were plated in 24-well plate chamber insert (Corning Life Sciences, Cat. 3422), with the medium containing 10% FBS at the bottom of the insert. Cells were incubated for 24 h, and then fixed with 4% paraformaldehyde for 20 min. After washing, cells were stained with 0.5% crystal violet blue. The positively stained cells were pictured and counted. Annexin V FITC Apoptosis Detection Kit I (556547, BD, China) was used to evaluate the cellular apoptosis according to the manufacturer’s instructions. For cell cycle analysis experiments, indicated cells were digested and washed with PBS twice and then fixed in 75% alcohol overnight at − 20 °C. The fixed cells were washed three times and then stained with propidium iodide (PI) staining buffer at room temperature for 30 min in the dark, and then the cell cycle was analysed by FACSAria SORP machine (BD, USA).

### Immunoprecipitation, immunoblot and Real-time RT-PCR assays

Briefly, cells were lysed in IP lysis buffer (1mM NaF, 50 mM Tris-HCl, pH 8.0, 120 mM NaCl, 0.5% NP40, 1 mM EDTA), supplemented with complete protease inhibitor cocktail (Complete Mini, Roche). To detect the physical interaction between indicated proteins, indicated constructs were transfected into HEK-293T cells, and indicated cell lysates were subjected to immunoprecipitation with indicated primary antibodies. The precipitated proteins were further detected with indicated antibodies by immunoblot. For cycloheximide (CHX) or MG132 treatment, indicated cells were treated with 100 μg/mL CHX, or 20 μM MG132 for 24 h. For Real-time RT-PCR assay, indicated cells were lysed by RNAiso Plus (Takara Bio, Beijing, China, Cat. 108-95-2). Total RNAs were extracted according to the manufacturer’s protocol, and then reverse transcribed using RT reagent Kit (Takara Bio, Beijing, China, Cat. RR047A; TIANGEN Biotech, Beijing, China, Cat. KR211-02). Real-time PCR was performed by FastStart Universal SYBR Green Master Mix (Roche, Cat. 04194194001; TIANGEN Biotech, Beijing, China, Cat. FP411-02) using an Applied Biosystems 7500 machine. The antibodies used in this study are shown in Table S2.

### Tumor growth assays in vivo

As previously documented [16], male nude mice aged 4-6 weeks were subcutaneously injected with indicated cells (1×10^6^ cells/point). At the end of the experiments, all mice were sacrificed and the tumors were harvested and weighted. Nude mice were monitored, xenograft tumor weights and volumes were measured with a sliding caliper, and tumor volumes were calculated using the formula (L×W^2^)/2. For ASO treatment assay, male nude mice aged 4-6 weeks were subcutaneously injected with A549 cells (1.5×10^6^ cells/point), when the xenograft tumors reached to 50 mm^3^ of volume, they were randomly divided into indicated groups, and the mice were injected with indicated ASOs (5nM) around the tumor twice per week. All animals were kept in a SPF environment and the protocols were pre-approved and conducted under the policy of Animal care and Use Committee at the Kunming Institute of Zoology, CAS.

### Dual-luciferase assay

Putative binding sites for miR-485-5p on the 3’-UTR of PKMYT1 and PKMYT1AR were predicted by StarBase (http://starbase.sysu.edu.cn/). Wild-type and mutant DNA fragments were sub-cloned into the luciferase reporter vector pGL3 (Promega). HEK-293T cells (2×10^4^ cells/well) were seeded in a 24-well plate and co-transfected with indicated plasmids using Lipofectamine 2000. Both firefly and Renilla luciferase expressions were measured post-transfection using the Dual Luciferase Kit (Promega) according to the manufacturer’s instructions.

### Immunohistochemical staining (IHC)

Briefly, the tissue sections were deparaffinized in xylene and rehydrated using graded ethanol. Antigen retrieval was performed for 20 min at 95 °C with sodium citrate buffer (pH 6.0). After quenching endogenous peroxidase activity with 3% H_2_O_2_ and blocking non-specific binding with 1% bovine serum albumin buffer, sections were incubated overnight at 4°C with indicated primary antibodies. Following several washes, the sections were treated with HRP conjugated secondary antibody for 40 min at room temperature, and stained with 3, 3-diaminobenzidine tetrahydrochloride (DAB). Slides were photographed with microscope (Olympus BX43F, Japan), and pictures were analyzed with the Image-Pro Plus 7.0 software (Media Cybernetics, Inc., Silver Spring, MD, USA). Samples were obtained with informed consent and all protocols were approved by The Second Xiangya Hospital of Central South University Ethics Review Board (Scientific and Research Ethics Committee, S-02/2000). Written informed consent was obtained from all patients. Fresh clinical samples were obtained from the Third Affiliated Hospital of Kunming Medical University in China (Table S6).

### Bioinformatics assay and statistical analysis

All datasets used in this study were available to the public. The expression of miRNAs, mRNAs in Gene Expression Omnibus (GEO) and TCGA dataset were obtained from the GEO website [17], TCGA official website [18] and StarBase [19], the datasets were analyzed by GEO2R. The survival analysis was performed according to the GEPIA website [20] and Kaplan-Meier Plotter [21]. KEGG pathway enrichment analysis was performed using the GSEA software [22]. The significance of the data between two experimental groups was determined by Student’s *t*-test, and multiple group comparisons were analyzed by one-way ANOVA. *P* < 0.05 (*), *P* < 0.01 (**) and *P* < 0.001 (***), were significant.

## Results

### PKMYT1AR is upregulated in NSCLC

**Fig. 1:**
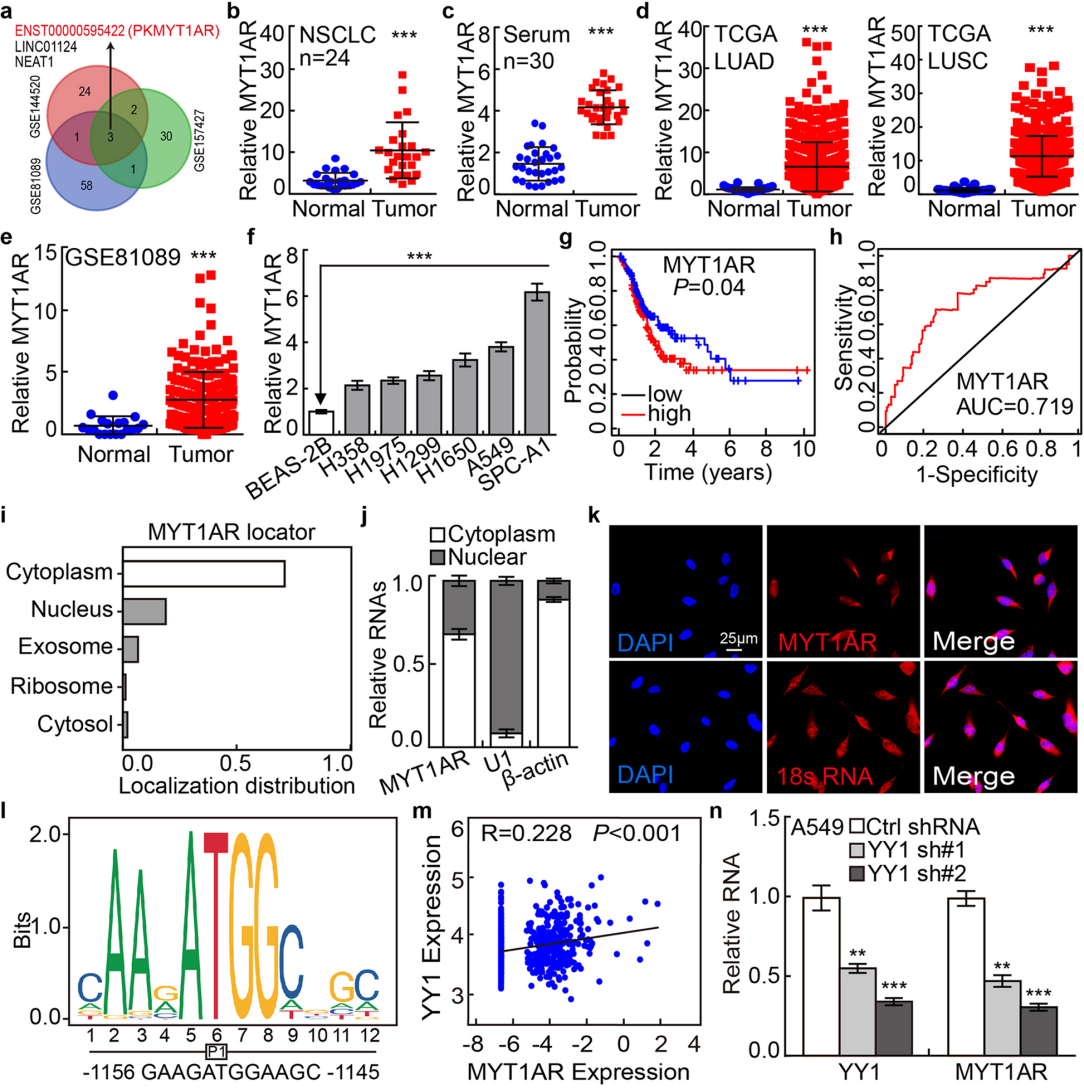
LncRNA PKMYT1AR is highly expressed in NSCLC. **a** LncRNA PKMYT1AR was identified by integrative analysis using GEO datasets, GSE81089 (Blue): Next Generation Sequencing (RNAseq) from NSCLC, GSE144520 (Red): whole-transcriptome sequencing of A549 cells and cisplatin-resistant A549/DPP cells, GSE157427 (Green): gene expression profile for lung cancer stem cells. **b** The relative expression level of PKMYT1AR in fresh paired tissues isolated from NSCLC patients using Real-time RT-PCR assay, *n*=24. **c** The expression of PKMYT1AR in paired NSCLC peripheral blood serum examined by the Real-time RT-PCR assay, *n*=30. **d** The relative expression level of PKMYT1AR in TCGA-LUAD (adenocarcinoma; Normal: 59; Tumor: 533) and TCGA-LUSC (squamous cell carcinoma; Normal: 49; Tumor: 502), respectively. **e** The relative expression pattern of PKMYT1AR in GSE81089 dataset (Normal: 19, Tumor: 197). **f** The relative expression level of PKMYT1AR in NSCLC cancerous cell lines, including H358, H1975, H1299, H1650, A549 and SPC-A1 examined by Real-time RT-PCR, compared to normal human bronchial epithelial cell line: BEAS-2B. **g** PKMYT1AR high expression correlates with worse survival rate. **h** The ROC curve for PKMYT1AR (AUC=0.719) in LUAD using TCGA dataset. **i** The subcellular localization of PKMYT1AR was predicted by LncLocater. **j** PKMYT1AR was majorly localized in the cytoplasm of A549 cells using nuclear and cytoplasmic RNA fractionation assay followed by Real-time RT-PCR. β-actin and U1 expressions were used as cytoplasmic and nuclear fraction controls, respectively. **k** The sub-cellular localization of PKMYT1AR was examined by FISH. The nuclei were stained with DAPI (blue), and the 18S RNA was used as cytoplasm-localized RNA control. Scale bar=25μm. **l** The YY1 binding motif within the PKMYT1AR promoter region was predicted using JASPAR. **m** The correlation between YY1 and PKMYT1AR were examined in TCGA-LUAD dataset. **n** Depletion of YY1 reduced PKMYT1AR expression in A549 cells examined by the Real-time RT-PCR assay. * *P* < 0.05, ** *P* < 0.01, *** *P* < 0.001. MYT1AR=PKMYT1AR

To identify critical lncRNAs involved in NSCLC progression, we examined the lncRNA expression profiles in NSCLC cancerous tissues, NSCLC cancerous cell lines including A549/DDP (cisplatinum resistant cell line) and cancer stem (or stem-like) cells, compared to reciprocal control groups. We found that 3 lncRNAs including PKMYT1AR, LINC01124 an NEAT1, were unanimously upregulated (Fig. 1a, Table S1). Except for PKMYT1AR, the other two lncRNAs have been well characterized in lung cancer [23–25]. PKMYT1AR, which is uniquely expressed in human but not other species and highly ranked [26], particularly drew our attention (Fig. S1a). Next, the upregulation of PKMYT1AR was verified in paired NSCLC cancerous tissues (*n*=24), and peripheral blood serum (*n*=30), using Real-time RT-PCR compared with reciprocal controls (Fig. 1b-c). Consistently, the upregulation of PKMYT1AR in NSCLC was verified using web available datasets (Fig. 1d-e) [27]. Furthermore, we found that PKMYT1AR was increased in NSCLC cancerous cell lines (H358, H1975, H1299, H1650, A549 and SPC-A1) compared with that in normal human bronchial epithelium cell line BEAS-2B (Fig. 1f). Importantly, PKMYT1AR high expression patients exhibit worse clinical outcome compared to the patients with lower PKMYT1AR expression (Fig. 1g). ROC curve analysis of PKMYT1AR showed an AUC value of 0.719, indicating its prognostic value in NSCLC (Fig. 1h). To verify whether PKMYT1AR is increased in CSCs, we then cultured A549 and SPC-A1 spheroid cells using culture condition favoring stem cell growth (Fig. S1b-c) [28]. In addition, PKMYT1AR was validated to be increased in A549/DDP cells compared with A549 cells (Fig. S1d). We also uncovered that PKMYT1AR was mainly localized in the cytoplasm of NSCLC cells using cellular fractionation assay followed by RNA fluorescence in situ hybridization (FISH), which was consistent with the online prediction dataset (Fig. 1i-k and Fig. S1e) [29]. In addition, we confirmed that PKMYT1AR could not be translated into coding-proteins using immunoblot (Fig. S1f-g).

The possible upstream factors of PKMYT1AR were screened using UCSC (http://genome.ucsc.edu/) and other bioinformatics dataset [30, 31], and multiple potential transcriptional factors were predicted (Fig. S1h). However, yin yang 1 (YY1) was identified as the only transcription factor for PKMYT1AR based on the positive correlation between YY1 and PKMYT1AR expressions in NSCLC, and the YY1 binding site in the promoter DNA sequence (P1) of PKMYT1AR was predicted using JASPAR (Fig. 1l-m) [30]. In line with previous documented studies [32], YY1 was confirmed to be highly expressed in NSCLC, and its high expression correlates with worse probability (Fig. S1i-j). To corroborate the regulatory role of YY1, we found that PKMYT1AR was decreased upon YY1 knockdown (Fig. 1n and Fig. S1k). These results suggest that YY1 induced PKMYT1AR may serve as a valuable prognostic biomarker for NSCLC.

### PKMYT1AR promotes tumor cell proliferation and migration

**Fig. 2:**
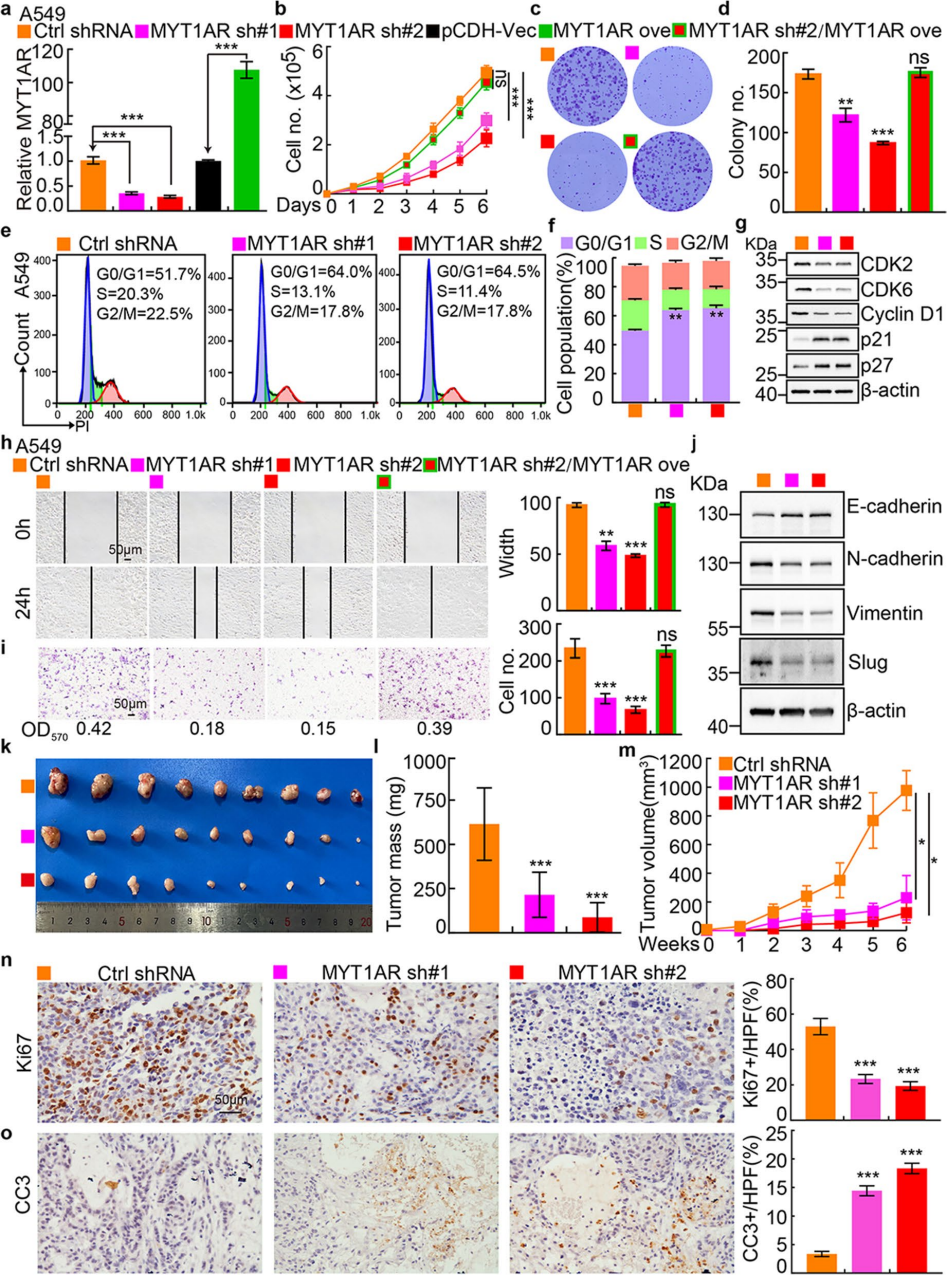
PKMYT1AR knockdown inhibits tumor cell growth and migration. **a** Establishment of PKMYT1AR overexpression and knockdown cell lines in A549 verified by Real-time RT-PCR. **b-d** PKMYT1AR knockdown dramatically inhibited A549 cell proliferation (**b**) and colony formation ability (**c**), (**d**) is the quantification data for (**c**). **e-f**. Effect of PKMYT1AR knockdown on the G0/G1 cell cycle transition was tested in A549 cells by PI staining and flow cytometry. **f** is the quantification data for (**e**). **g** PKMYT1AR knockdown regulated the expressions of cell cycle transition mediators, including CDK2, CDK6, cyclin D1, p21 and p27. Indicated cell extracts were probed with indicated antibodies. **h-i** Knockdown of PKMYT1AR inhibited A549 cell migration using wound healing (**h**) and transwell (**i**) assays. Quantification data were also indicated, and the OD_570_ values for trans-well assay were indicated below. Scale bar= 50 μm. **j** Indicated cell extracts were probed with indicated antibodies to examine the expression patterns of cell migration regulators, including E-cadherin, N-cadherin, Vimentin and Slug. **k-m** PKMYT1AR knockdown inhibited xenograft tumor formation in vivo. Representative xenograft tumor images (**k**), tumor masses (**l**) and tumor volumes (**m**) were shown. **n-o** Representative IHC staining of Ki67 (**n**) and Cleaved Caspase 3 (CC3, **o**) for indicated xenograft tumors. Quantification data were also indicated. Scale bar=50 μm. * *P* < 0.05, ** *P* < 0.01, *** *P* < 0.001. HPF=high power field, pCDH-Vec=pCDH lenti-viral plasmid vector control. ove=over-expression, sh#1=shRNA#1, sh#2=shRNA#2

To examine the functional role of PKMYT1AR in tumor cells, potential molecular events associated with PKMYT1AR were analyzed using GSEA dataset [22], and signaling pathways including cell cycle checkpoint and epithelial-mesenchymal transition (EMT) were identified (Fig. S2a). To corroborate these correlations, PKMYT1AR was inhibited by two independent lenti-viral shRNAs in A549 and SPC-A1 cells, and the knockdown efficiencies were verified by Real-time RT-PCR, cell line expressing scramble shRNA was used as control (Materials and methods, Fig. 2a and Fig. S2b, Table S2). As expected, PKMYT1AR knockdown inhibited A549 and SPC-A1 cell proliferation, whereas PKMYT1AR forced expression markedly overcame this phenotype (Fig. 2b and Fig. S2c). Furthermore, the colony formation ability was also markedly decreased in PKMYT1AR knockdown cells compared with control shRNA cells (Fig. 2c-d and Fig. S2d-e). We then investigated the cell cycle transition by flow cytometry analysis and identified that PKMYT1AR knockdown resulted in accumulated G0/G1 phase cell population in A549 and SPC-A1 (Fig. 2e-f and Fig. S2f). In addition, CDK2, CDK6 and Cyclin D1, the key regulators for G0/G1 cell cycle checkpoint were markedly decreased, while p21 and p27 were increased in PKMYT1AR depleted cells examined by immunoblot (Fig. 2g and Fig. S2g). Next, we used wound healing and trans-well assays to test whether PKMYT1AR regulates tumor cell migration, and the results showed that the tumor cell migration ability was dramatically repressed in PKMYT1AR knockdown cells compared with control group (Fig. 2h-i and Fig. S2h-i). The expressions of marker genes important for EMT were also examined by immunoblot. As expected, E-cadherin was increased, while N-cadherin, vimentin and slug were decreased after PKMYT1AR inhibition (Fig. 2j and Fig. S2j). We also uncovered that PKMYT1AR knockdown promoted cellular apoptosis (Fig. S2k-l). The above results supported the oncogenic role of PKMYT1AR in NSCLC.

To further define the functional role of PKMYT1AR regulating NSCLC progression in vivo, we performed the xenograft tumor formation assay. Male nude mice of 5 weeks old age were randomly divided into 3 groups, A549 cells stably expressing control shRNA or PKMYT1AR-targeting shRNAs, were injected subcutaneously (1x10^6^ cells/point), and the tumor growth and volumes were monitored every week. Consistent with the results in vitro, PKMYT1AR knockdown markedly retarded tumor progression in vivo (Fig. 2k-m). The cell proliferation marker Ki67 and cellular apoptosis marker cleaved caspase 3 (CC3) in the xenograft tumors were then examined by immunohistochemical (IHC) staining, and we verified that PKMYT1AR knockdown inhibited tumor cell proliferation but promoted cellular apoptosis in vivo (Fig. 2n-o).

### PKMYT1AR functions as a ceRNA for miR-485-5p to promote PKMYT1 expression

**Fig. 3:**
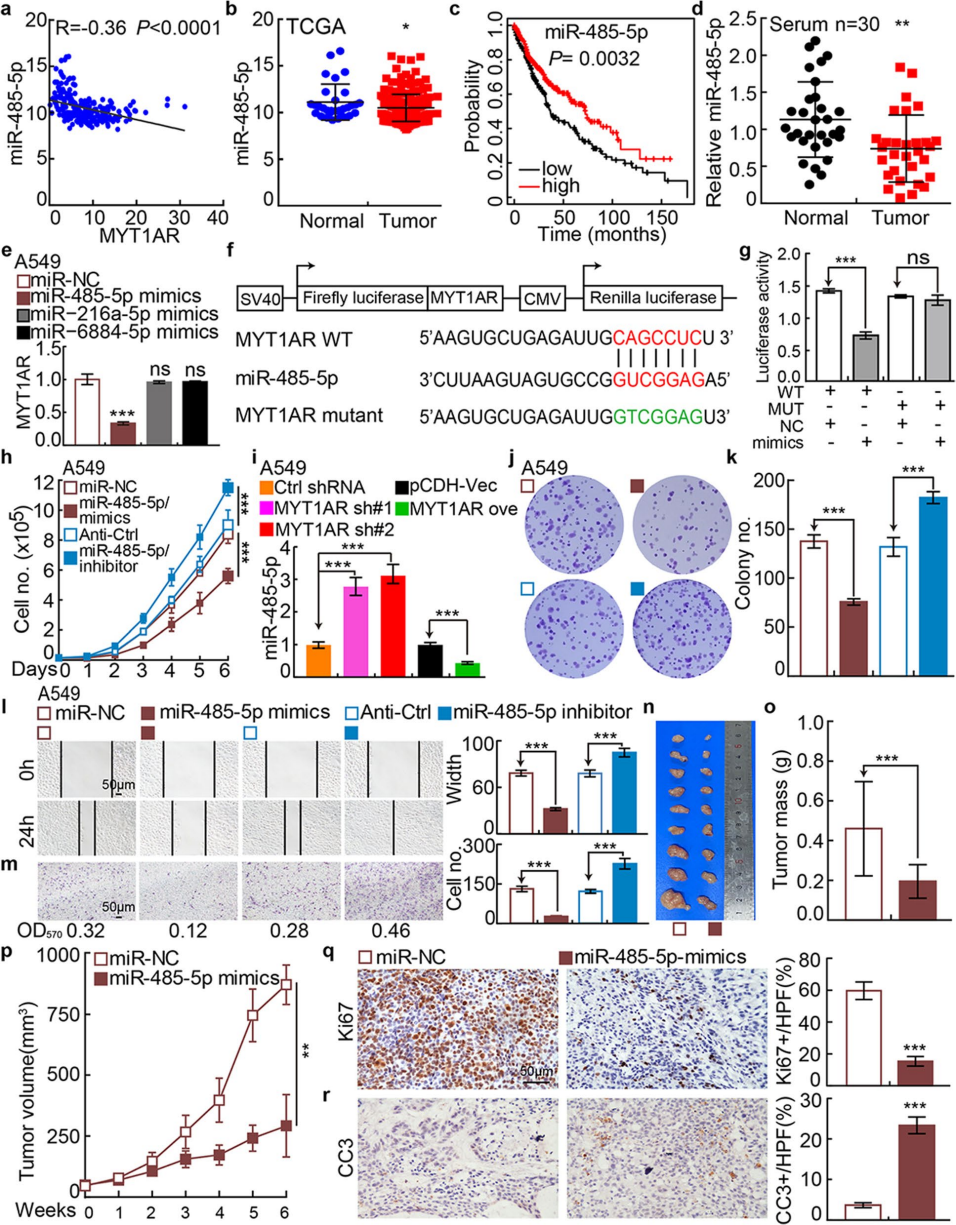
miR-485-5p inhibits tumor progression. **a** Correlation analysis between PKMYT1AR and miR-485-5p using TCGA-LAUD dataset. **b** The decreased expression of miR-485-5p in TCGA-LAUD dataset. **c** The prognostic value of miR-485-5p in TCGA-LAUD examined by Kaplan-Meier Plotter. **d** The expression of miR-485-5p in fresh peripheral blood serum samples isolated from normal and NSCLC patients, respectively, verified by Real-time RT-PCR. **e** The expression of PKMYT1AR after over-expression of miR-485-5p, miR-216a-5p and miR-6884-5p in A549 cells examined by Real-time RT-PCR. **f** A schematic picture of the wild-type (WT) and mutant (MUT) PKMYT1AR luciferase reporter plasmids. **g** The luciferase activities of the PKMYT1AR luciferase reporters (WT or MUT) were examined in HEK-293T cells with miR-485-5p mimics or mimic NC co-expression. **h** miR-485-5p regulated A549 cell proliferation assay. **i** Relative miR-485-5p expression examined by Real-time RT-PCR assay in indicated cells. **j-k** miR-485-5p regulated A549 colony formation assay (**j**), (**k**) is the quantification data for (**j**). **l-m** miR-485-5p regulated A549 cell migration examined by wound healing (**l**) and trans-well (**m**) assays. Quantification data were also presented, and the OD_570_ values for trans-well assay were indicated below. Scale bar=50 μm. **n-p** Representative xenograft tumor images (**n**), tumor masses (**o**) and tumor volumes (**p**) were shown for indicated groups. A549 cells were used. **q-r** Representative IHC staining of Ki67 (**q**) and CC3 (**r**) for indicated xenograft tumors. Quantification data were also indicated. Scale bar=50 μm. * *P* < 0.05, ** *P* < 0.01, *** *P* < 0.001. mimics=miR-485-5p mimics, miR-NC=NC=miRNA mimics control, Anti-Ctrl=miRNA inhibitor control, WT=PKMYT1AR wild-type luciferase reporter, MUT=PKMYT1AR mutant luciferase reporter

To explore the molecular mechanism by which PKMYT1AR promotes NSCLC progression, we used StarBase to predict the potential microRNAs directly interacting with PKMYT1AR [19], and multiple candidate microRNAs including miR-216a-5p, miR-485-5p and miR-6884-5p were identified. To validate the specific PKMYT1AR targeting miRNA(s), the expressions of the candidate miRNAs and their correlation with PKMYT1AR in NSCLC were examined [19, 33]. We found that miRNA-485-5p, but not miR-216a-5p and miR-6884-5p, was negatively associated with PKMYT1AR expression, and its lower expression in NSCLC correlates with worse clinical outcome (Fig. 3a-c and Fig. S3a-b). In addition, consistent with web source database [19], miR-485-5p was validated to be downregulated in the peripheral blood serum isolated from NSCLC patients (Fig. 3d). Importantly, only miR-485-5p forced expression could inhibit PKMYT1AR expression, indicating the specific association between PKMYT1AR and miR-485-5p in NSCLC (Fig. 3e and Fig. S3c-d).

The 3’-UTR binding site of miR-485-5p in PKMYT1AR was identified [19] and the luciferase activity of wild-type but not the mutant PKMYT1AR 3’-UTR containing reporter construct was inhibited by miR-485-5p mimics overexpression (Fig. 3f-g). miR-485-5p mimics or inhibitor with reciprocal controls were then overexpressed in A549 and SPC-A1 cells, and we found that the proliferation, colony formation and migration abilities of cancerous cells were abrogated by miR-485-5p mimics, while were upregulated by miR-485-5p inhibitor (Fig. 3h-m and Fig. S3e-j). As expected, miR-485-5p mimics overexpression markedly reduced tumor growth in vivo (Fig. 3n-r). Furthermore, the reduced cell proliferation, colony formation and migration abilities of tumor cells caused by PKMYT1AR knockdown can be overcame by miR-485-5p inhibitor overexpression, strongly supporting the specific role of PKMYT1AR/ miR-485-5p axis (Fig. S3k-p).

**Fig. 4:**
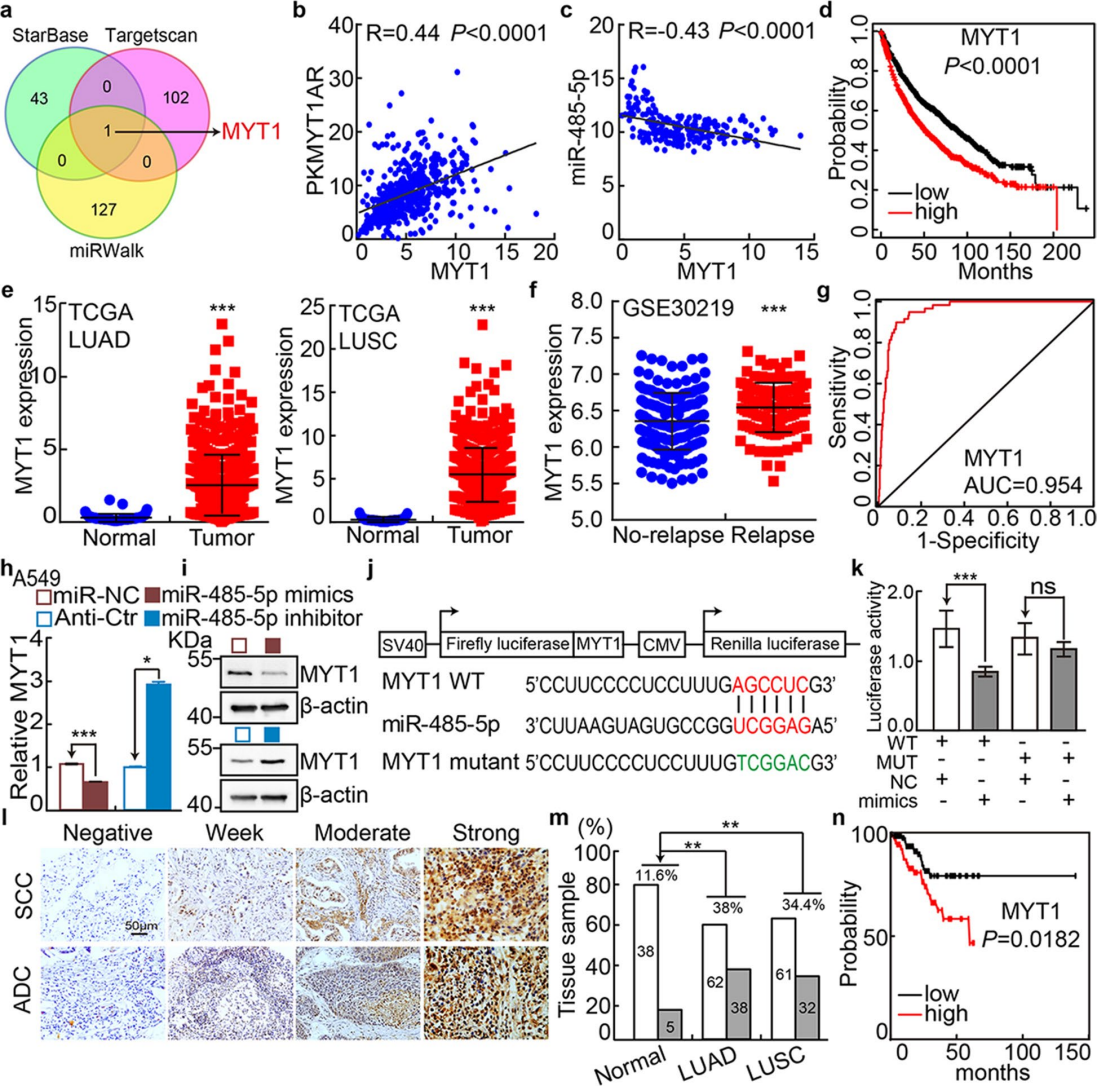
PKMYT1 is targeted by miR-485-5p and highly expressed in NSCLC. **a** Identifying PKMYT1 as the downstream target of miRNA-485-5p using various datasets (Green: StarBase; Pink: Targetscan; Yellow: miRWalk), and the top 50% of the target genes were selected. **b** The positive correlation between PKMYT1AR and PKMYT1 expressions in TCGA-LAUD dataset. **c** The negative correlation between miRNA-485-5p and PKMYT1 expressions in TCGA-LAUD dataset. **d** The prognostic value of PKMYT1 in TCGA-LAUD examined by Kaplan-Meier Plotter. **e** The relative expression level of PKMYT1 in TCGA-LUAD (Normal: 59; Tumor: 533) and TCGA-LUSC (Normal: 49; Tumor: 502), respectively. **f** The relative expression of PKMYT1 in GSE30219 datasetset (No-relapse:164; Relapse:114). **g** The ROC curve for PKMYT1 (AUC=0.954) in LUAD using TCGA dataset. **h-i** miR-485-5p mimics or inhibitor suppressed or promoted, respectively, PKMYT1 expression in A549 cells tested by Real-time RT-PCR (**h**) and immunoblot (**i**). **j** A schematic graph of the wild-type (WT) and mutant (MUT) PKMYT1 3’-UTR containing luciferase reporter plasmids. **k** The luciferase activities of the PKMYT1 3’-UTR containing luciferase reporters (WT or MUT) were examined in HEK-293T cells with miR-485-5p mimics or mimic NC co-expression. **l-m** IHC staining of PKMYT1 proteins in NSCLC cancerous tissues, **m** is the quantification data for (**l**). Scale bar=50 μm. **n** PKMYT1 high expression correlates with worse overall survival using IHC data from tissue microarray (**l**-**m**). * *P* < 0.05, ** *P* < 0.01, *** *P* < 0.001

We then tried to identify the direct downstream genes targeted by miR-485-5p using multiple web source available datasets, based on the following screening conditions, 1) the negative correlation between miR-485-5p and its targeting gene should be identified, 2) and the direct downstream gene should positively correlate with lncRNA-ENST00000595422 in NSCLC [19, 34, 35]. PKMYT1 was the only shared common gene among all the dataset, which positively, but negatively, associates with PKMYT1AR and miR-485-5p expressions in NSCLC, respectively (Fig. 4a-c, Table S3). Consistent with former findings [14, 36], PKMYT1 is highly expressed and frequently mutated in multiple types of human cancers including NSCLC, which results in worse clinical outcome (Fig. 4d-g and Fig. S4a-c, Table S4-S5). In addition, we found that miR-485-5p mimics overexpression decreased PKMYT1 transcript and protein expressions in NSCLC cancerous cell lines, which was validated by luciferase reporter assay, Real-time RT-PCR and immunoblot (Fig. 4h-k and Fig. S4d-e). Therefore, we decided to name the newly identified lncRNA-ENST00000595422 as PKMYT1AR.

**Fig. 5:**
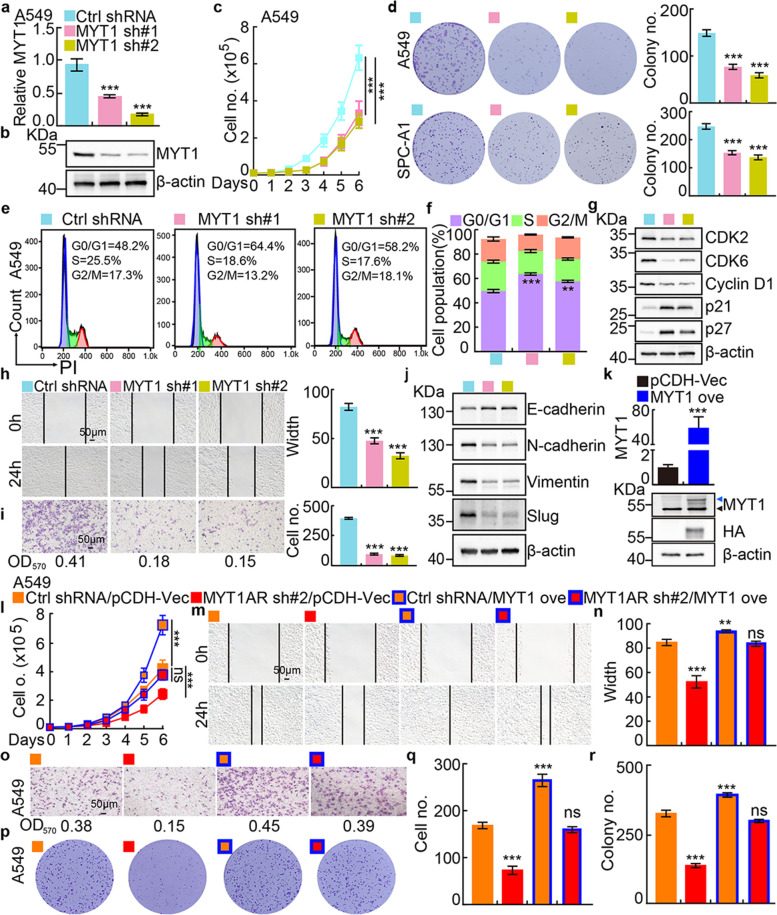
Depletion of PKMYT1 inhibits tumor growth. **a-b** Establishment of PKMYT1 knockdown in A549 cells, verified by Real-time RT-PCR (**a**) and immunoblot (**b**). **c** PKMYT1 knockdown dramatically inhibited A549 cell proliferation using growth curve assay. **d** PKMYT1 knockdown inhibited colony formation ability of A549 and SPC-A1 cells, respectively. Quantification data were also indicated. **e-f** Effect of PKMYT1 knockdown on the G0/G1 cell cycle transition was examined in A549 cells by PI staining and flow cytometry. **f** is the quantification data for (**e**). **g** PKMYT1 knockdown regulated the expressions of cell cycle transition mediators, including CDK2, CDK6, cyclin D1, p21 and p27. Indicated cell extracts were probed with indicated antibodies. **h-i** PKMYT1 regulated A549 cell migration examined by wound healing (**h**) and trans-well (**i**) assays. Quantification data were also presented, and the OD_570_ values for trans-well assay were indicated below. Scale bar=50 μm. **j** Indicated cell extracts were probed with indicated antibodies to examine the expression patterns of cell migration regulators, including E-cadherin, N-cadherin, Vimentin and Slug. **k** Validation of PKMYT1 over-expression by Real-time RT-PCR (top) and immunoblot (bottom) assays. Blue arrow head: exogenous HA-tagged PKMYT1; black arrow head: endogenous PKMYT1. **l–r** PKMYT1 forced expression overcame PKMYT1AR knockdown effect by cell growth curve (**l**), wound healing (**m-n**), trans-well (**o**, **q**) and colony formation assays (**p**, **r**), (**n**, **q**, **r**) were quantification data for reciprocal assays. Scale bar=50 μm. * *P* < 0.05, ** *P* < 0.01, *** *P* < 0.001. MYT1 ove=PKMYT1 over-expression

We further surveyed the protein expression and cellular location of PKMYT1 in NSCLC (including lung SCC and ADC) and noncancerous control lung tissues (NCLT) by immunohistochemical staining (IHC) using tissue microarray and immunoblot using fresh tissues. The percentage of positive PKMYT1 expression was significantly higher in NSCLC tissues than that in NCLT tissues (Fig. 4l-n and Fig. S4j). Consistently, PKMYT1 was identified to be highly expressed in NSCLC cancerous cell lines (Fig. S4f-i), and knockdown of PKMYT1 inhibited the cell proliferation, colony formation and migration abilities of tumor cells (Fig. 5a-j and Fig. S4k, S5a-i). The rescue experiment was performed to validate the specificity of PKMYT1AR/PKMYT1 axis, and PKMYT1 forced expression could overcome the cellular effect resulted from PKMYTAR knockdown (Fig. 5k-r and Fig. S5j-q). We also found that PKMYT1 knockdown promoted cellular apoptosis (Fig. S5r-s).

### PKMYT1AR/miR-485-5p/PKMYT1 axis promotes cancer stem cell maintenance in NSCLC

**Fig. 6:**
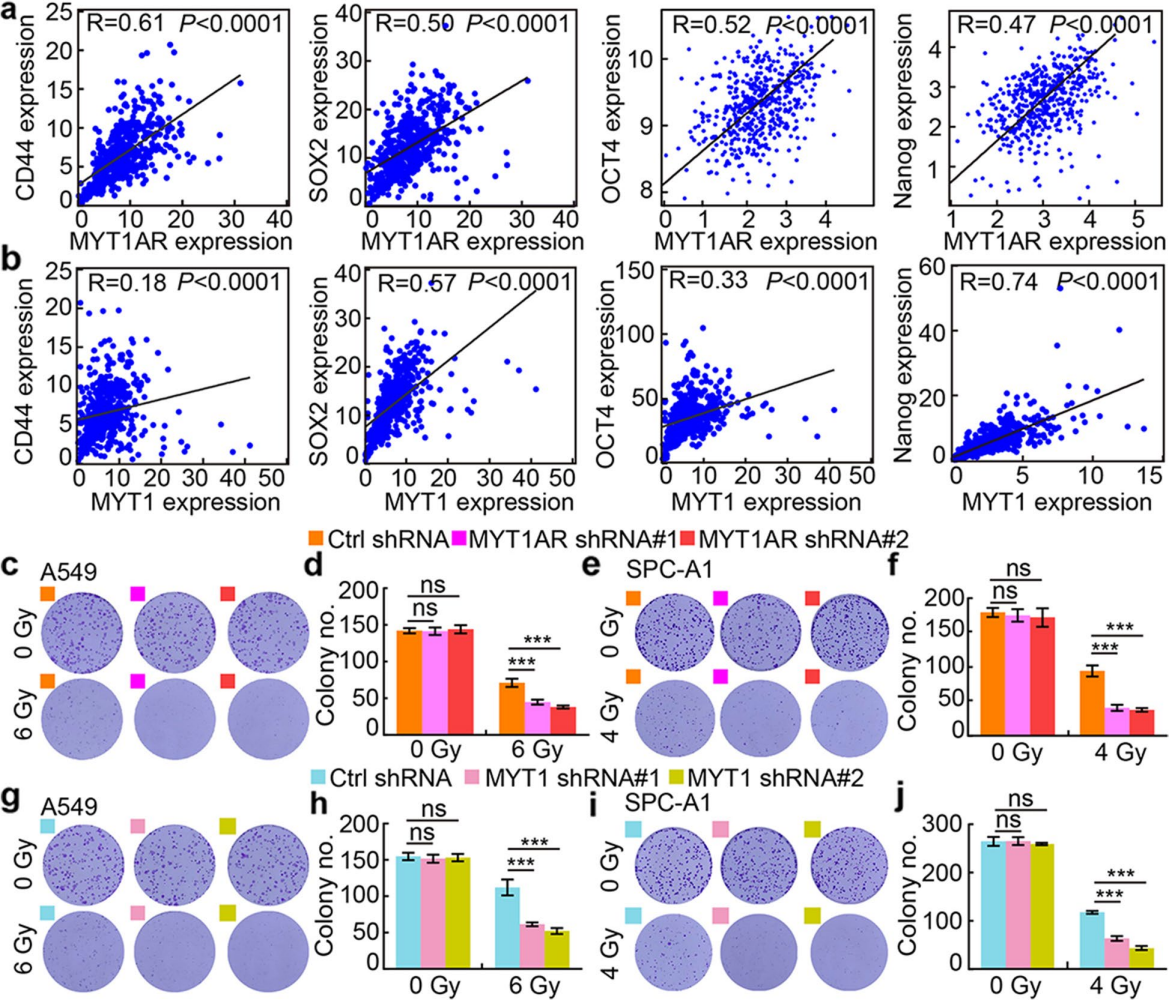
PKMYT1AR knockdown promotes tumor cell response to DDP. **a-b** The positive correlations between PKMYT1AR (**a**), or PKMYT1 (**b**), and stem cell maintenance related genes, including CD44, SOX2, OCT4 and Nanog, were examined using TCGA-LUAD dataset by Pearson’s correlation analysis. **c-f** Representative images of colony formation in indicated cells exposed to 0, 4, 6 Gy of X-ray irradiation. **d**, **f** were quantification data for indicated assays. **g-j** Representative images of colony formation in indicated cells exposed to 0, 4, 6 Gy of X-ray irradiation. **h**, **j** were quantification data for indicated assays. * *P* < 0.05, ** *P* < 0.01, *** *P* < 0.001

To further characterize the molecular mechanism by which PKMYT1AR/miR-485-5p/PKMYT1 axis regulates NSCLC progression, we performed the co-expression analysis and found that PKMYT1AR or PKMYT1 positively, whereas miR-485-5p negatively, correlates with the expressions of well defined cancer stem cell marker genes, including CD44, Sox2, Oct4, Nanog and ALDH1 (Fig. 6a-b and Fig. S6a) [11]. Based on the fact that the existence of CSCs is important for chemo- or radio-therapy resistance, we performed cell viability and irradiation sensitivity assays, and collected indicated cells for annexin V staining followed by flow cytometry analysis with or without cisplatin treatment. Indeed, less survived cells were detected upon DDP and Gy X-ray irradiation treatments, and increased apoptotic cell population was detected upon PKMYT1AR or PKMYT1 knockdown compared to control groups (Fig. 6c-j and Fig. S6b-c, e-f), which was further confirmed by immunoblot examining marker gene expressions critical for cellular apoptosis, including Bax, Bcl-2 and PARP (Fig. S6d, g). In line with the in vitro finding, PKMYT1AR inhibition sensitized tumor cells responding to DDP in vivo by xenograft tumor formation assay, while PKMYT1 forced expression could reverse PKMYT1AR knockdown effect, indicating the specific role of PKMYT1AR/miR-485-5p/PKMYT1 axis during NSCLC progression in vivo (Fig. S6h-m).

**Fig. 7:**
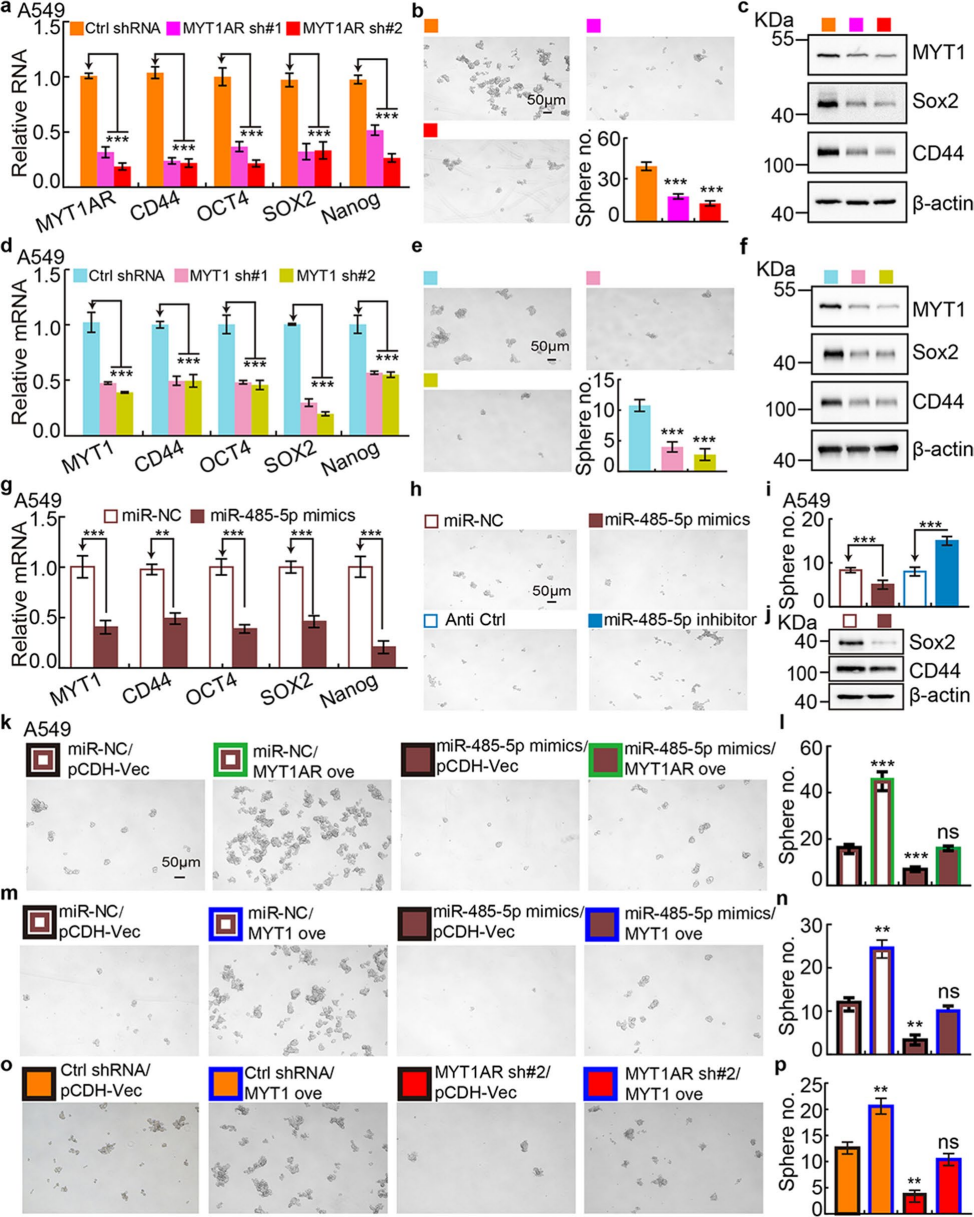
Depletion of PKMYT1AR inhibits cancer stem cell maintenance. **a** Relative mRNA expressions of PKMYT1AR and cancer stem cell marker genes, including CD44, OCT4, SOX2, Nanog in A549 cells, were examined by Real-time RT-PCR upon PKMYT1AR knockdown. **b** Tumor sphere formation abilities of indicated cells after PKMYT1AR knockdown were examined. Scale bar=50 μm. **c** Total extracts of indicated cells were probed with indicated antibodies by immunoblot. **d** Relative mRNA expressions of PKMYT1 and cancer stem cell marker genes in A549 cells, were examined by Real-time RT-PCR upon PKMYT1 knockdown. **e** Tumor sphere formation abilities of indicated cells after PKMYT1 knockdown were examined. Scale bar=50 μm. **f** Total cell extracts of indicated cells were probed with indicated antibodies by immunoblot. **g** Relative mRNA expressions of PKMYT1 and cancer stem cell marker genes in A549 cells, were examined by Real-time RT-PCR with miR-485-5p mimics or miR-NC co-expression. **h-i** Tumor sphere formation abilities of indicated cells after miR-485-5p mimics or miRNA controls co-transfection were examined (**h**) in A549 cells. **i** is the quantification data for (**h**). Scale bar=50 μm. **j** Total extracts of indicated cells were probed with indicated antibodies by immunoblot. **k-l** Rescue effect of PKMYT1AR over-expression on miR-485-5p mimics-mediated phenotype was examined by tumor sphere formation assay (**k**), **l** is the quantification data for (**k**). Scale bar=50 μm. **m-n** Rescue effect of PKMYT1 over-expression on miR-485-5p mimics-mediated phenotype was examined by tumor sphere formation assay (**m**), (**n**) is the quantification data for (**k**). **o-p** Rescue effect of PKMYT1 over-expression on PKMYT1AR depletion-mediated phenotype was examined by tumor sphere formation assay (**o**), (**p**) is the quantification data for (**o**). * *P* < 0.05, ** *P* < 0.01, *** *P* < 0.001

To corroborate the hypothesis that PKMYT1AR/miR-485-5p/PKMYT1 axis is critical for CSCs maintenance, cancer stem (like) cells were obtained using spheroid culture condition [28]. The mRNA and protein expressions of CSCs marker genes, including CD44, Sox2, Oct4 and Nanog, were examined, which were markedly decreased after PKMYT1AR or PKMYT1 knockdown (Fig. 7a, c, d, f, g, j and Fig. S7a, c, d, f, g, j). Furthermore, we revealed that knockdown of PKMYT1AR or PKMYT1, but overexpression of miR-485-5p, inhibited the tumor sphere formation ability (Fig. 7b, e, h-i and Fig. S7b, e, h-i). Most importantly, the reciprocal rescue experiment results based on the signaling axis of PKMYT1AR/miR-485-5p/PKMYT1 using tumor sphere formation assay, strongly support the specific role in NSCLC cancer stem cells (Fig. 7k-p and Fig. S7k-p).

### PKMYT1 activates Wnt signaling through stabilizing β-catenin proteins

**Fig. 8:**
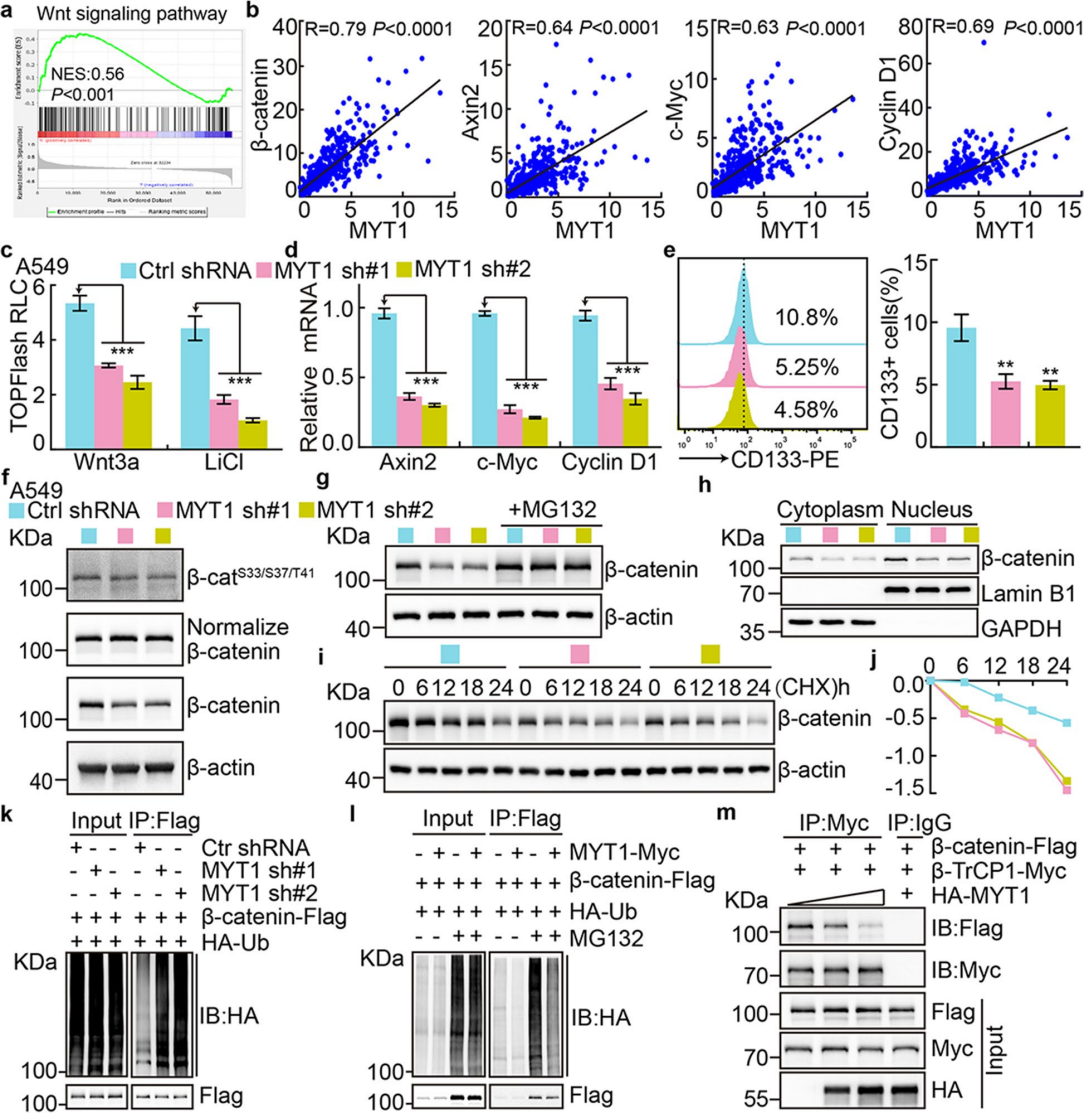
PKMYT1 activates Wnt signaling. **a** Wnt signaling pathway was enriched by GSEA analysis. **b** The correlations between PKMYT1 and Wnt signaling factors, including β-catenin, Axin2, c-Myc and Cyclin D1 in TCGA-LUAD , were examined using pearson’s correlation analysis. **c** Wnt signaling pathway activity was examined using TOPFlash reporter assay in indicated cells after Wnt3a or LiCl (100 μM) treatment, respectively. **d** Relative expression of indicated transcripts were examined by Real-time RT-PCR upon PKMYT1 knockdown. **e** Membrane-tethered form of cancer stem cell marker CD133 was examined in indicated cells by flow cytometry assay. Quantification result was indicated (right). **f** Total and phosphorylated forms of β-catenin proteins were examined by immunoblot with indicated antibodies in A549 cells. β-catenin Phosphorylation level was detected after normalization of endogenous β-catenin proteins, but not β-actin. **g** Total β-catenin proteins were reduced after PKMYT1 knockdown in A549 cells, which can be reversed by MG132 (20 μM) treatment. **h** PKMYT1 knockdown inhibited nuclear accumulation of β-catenin proteins in A549 cells by immunoblot. Lamin B: nuclear fraction; GAPDH: cytosol fraction. **i-j** Lysates of indicated cells treated by cycloheximide (CHX: 100 μg/mL) were examined by immunoblot with indicated antibodies. (**j**) is the quantification data for (**i**). **k-l** Examining the ubiquitination levels of β-catenin proteins upon PKMYT1 knockdown (**k**) or over-expression (**l**) by immunoblot using indicated antibodies under different co-transfection conditions. **m** Co-IP assay was used to detect the association among PKMYT1, β-TrCP1 and β-catenin in HEK-293T cells. Myc-tagged and HA-tagged PKMYT1 were used for different assays. * *P* < 0.05, ** *P* < 0.01, *** *P* < 0.001

To identify the downstream factors mediating the functional role of PKMYT1 in NSCLC, we performed the KEGG and co-expression analysis, and uncovered that PKMYT1 were tightly associated with Wnt signaling pathway (Fig. 8a-b) [22]. The TOP Flash luciferase reporter assay confirmed that PKMYT1 knockdown decreased the canonical Wnt signaling activated by Wnt3a ligand and LiCl [37]. Furthermore, the Wnt signaling downstream targeted genes, including Axin2, c-Myc and cyclin D1 were also decreased (Fig. 8c-d and Fig. S8a-b). In addition, the membrane tethered form of CD133 expression, a documented biomarker for cancer stem cell [38], was markedly reduced upon PKMYT1 inhibition (Fig. 8e and Fig. S8c). Canonical Wnt signaling is activated after blocking β-catenin phosphorylation mediated by GSK-3β leading to its stabilization and translocation into the nucleus [39], we then examined the phosphorylation pattern and stability of β-catenin proteins by immunoblot in PKMYT1 depleted cells compared to control cells. We did not detect significant change on Serine 33/37 and Threonine 41 sites mediated by GSK-3β by immunoblot after PKMYT1 knockdown (Fig. 8f and Fig. S8d). To our surprise, dramatic reduced β-catenin proteins in both cytoplasmic and nucleus were detected compared to control group, which was reversed by MG132 treatment, a proteasome inhibitor (Fig. 8g-h and Fig. S8e-f). We also examined the β-catenin protein degradation rate, and found that depletion of PKMYT1 accelerated β-catenin degradation in A549 and SPC-A1 cells (Fig. 8i-j and Fig. S8g-h). Consistently, we found that exogenous ubiquitinated β-catenin proteins were markedly increased or decreased by depleting or overexpressing PKMYT1, respectively (Fig. 8k-l).

Since β-catenin phosphorylation by GSK-3β creates a binding site for the E3-ligase SCF^β-TrCP^, leading to β-catenin ubiquitin-proteasome degradation [40], we decided to examine whether the E3-ligase recruitment could be depleted in the presence of high expression of PKMYT1. By co-immunoprecipitation assay, we found that both endogenous and exogenous β-catenin bound to PKMYT1 or β-TrCP1, respectively, while the exogenous β-catenin/β-TrCP1 complex formation was abrogated by PKMYT1 overexpression in a dosage dependent manner (Fig. 8m and Fig. S8i-l). The above results suggest that PKMYT1 reduces β-catenin degradation by blocking E3-ligase SCF^β-TrCP^ binding to β-catenin, leading to the constitutive activation of Wnt signaling and self-renewal of cancer stem cells.

### The therapeutic effect by targeting PKMYT1AR with ASO

**Fig. 9:**
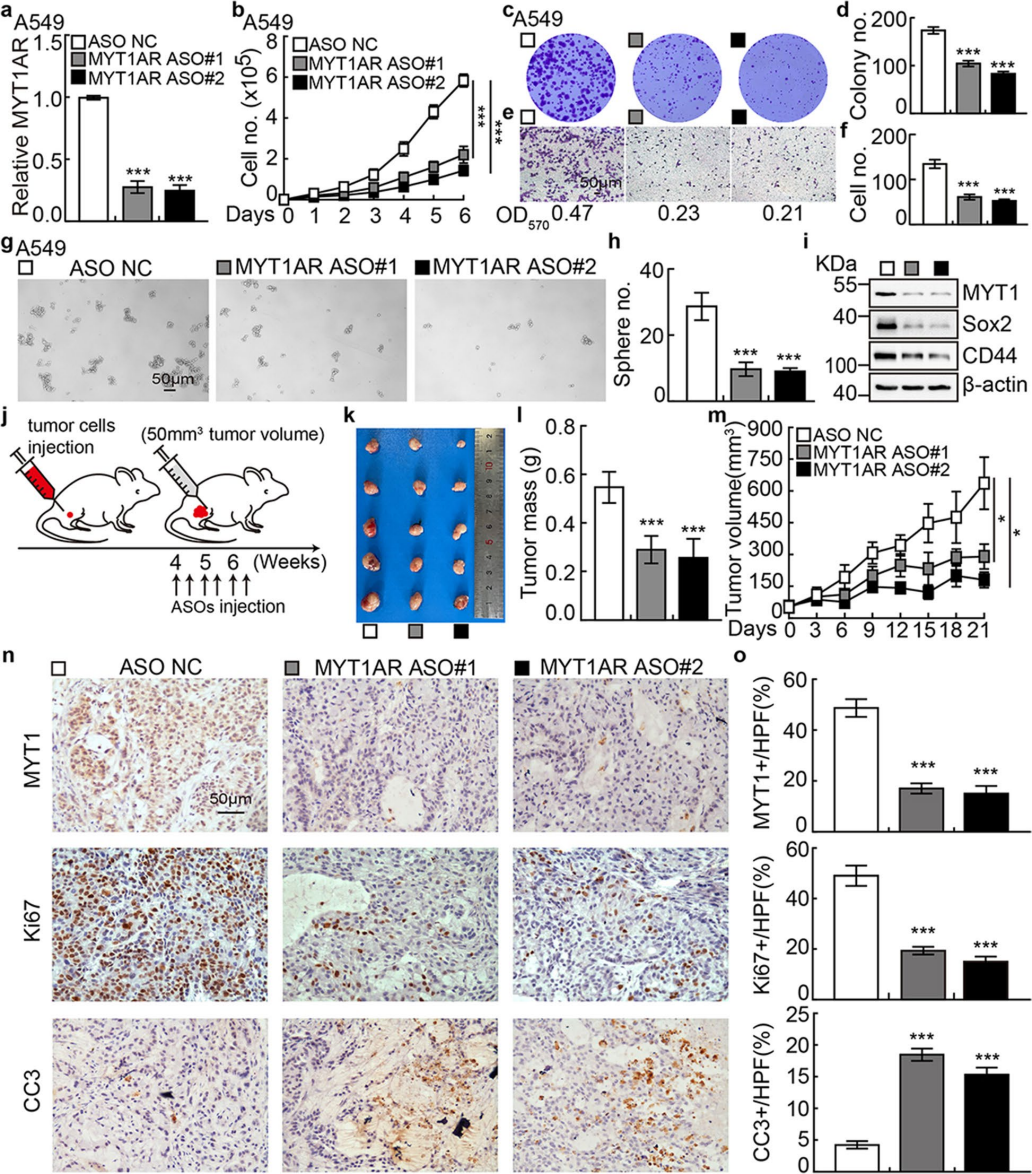
PKMYT1AR targeting ASOs impede tumor growth. **a** The relative expressions of PKMYT1AR transcripts were examined by Real-time RT-PCR after indicated ASO transfection. **b** Knockdown of PKMYT1AR inhibited cell proliferation examined by growth curve assays. **c-d** PKMYT1AR targeting ASOs repressed cell growth by colony formation assay. **d** is the quantification data for (**c**). **e-f** PKMYT1AR targeting ASOs repressed cell migration by trans-well assay (**e**). **f** is the quantification data for **e**. Scale bar=50 μm. **g-h** PKMYT1AR targeting ASOs inhibited cancer stem cell self-renewal ability by tumor-sphere formation assay (**g**). **h** is the quantification data for (**g**). Scale bar=50 μm. **i** PKMYT1AR targeting ASOs inhibited cancer stem cell marker genes expressions examined by immunoblot with indicated antibodies. **j** Schematic view of xenograft mouse model treated by indicated ASOs. **k-m** Representative xenograft tumor images (**k**), tumor masses (**l**) and tumor volumes (**m**) were shown for indicated groups treated by indicated ASOs. A549 cells were used. **n-o** Representative IHC staining of PKMYT1, Ki67 and CC3 (**n**) for indicated xenograft tumors. **o** is the quantification data for (**n**). Scale bar=50 μm. * *P* < 0.05, ** *P* < 0.01, *** *P* < 0.001. ASO NC=ASO control

Antisense oligonucleotide (ASO) drugs have been demonstrated to be effective on inhibiting tumor growth both in vitro and in vivo [41]. To verify whether PKMYT1AR could be inhibited by ASO, two ASOs specifically targeting PKMYT1AR and control ASO were designed (Table S2). PKMYT1AR transcript was markedly repressed by both targeting ASOs in A549 and SPC-A1 cells compared to control ASO treatment (Fig. 9a and Fig. S9a). Cell proliferation, migration, and CSCs self-renewal abilities were also inhibited upon PKMYT1AR targeting ASOs treatment compared with control group (Fig. 9b-h and Fig. S9b-h).

Consistently, the protein expressions of PKMYT1, β-catenin, Sox2 and CD44, were significantly decreased in PKMYT1AR ASOs interfering groups compared to control group (Fig. 9i and Fig. S9i). Xenograft tumor formation mouse model was also applied to evaluate the therapeutic efficacy of PKMYT1AR ASOs treatment. Wild type A549 cells were inoculated subcutaneously in nude mice, after 4 weeks until the xenograft tumors reaching about 50mm^3^, mice were randomly divided into three groups (ASO-control, PKMYT1AR ASO#1, PKMYT1AR ASO#2), and then given different ASOs, respectively by injection around xenograft tumors twice a week. Significant reduction of tumor growth visualized by decreased Ki67 staining and PKMYT1 expression, but increased CC3 expression, were detected in PKMYT1AR ASOs groups compared to control group (Fig. 9j-o). As expected, the expressions of CSCs marker gene CD44, Sox2 and β-catenin were decreased in PKMYT1AR targeting ASOs groups, indicating the promising therapeutic effect treating NSCLC patients in the future (Fig. S9j-k).

## Discussion

**Fig. 10:**
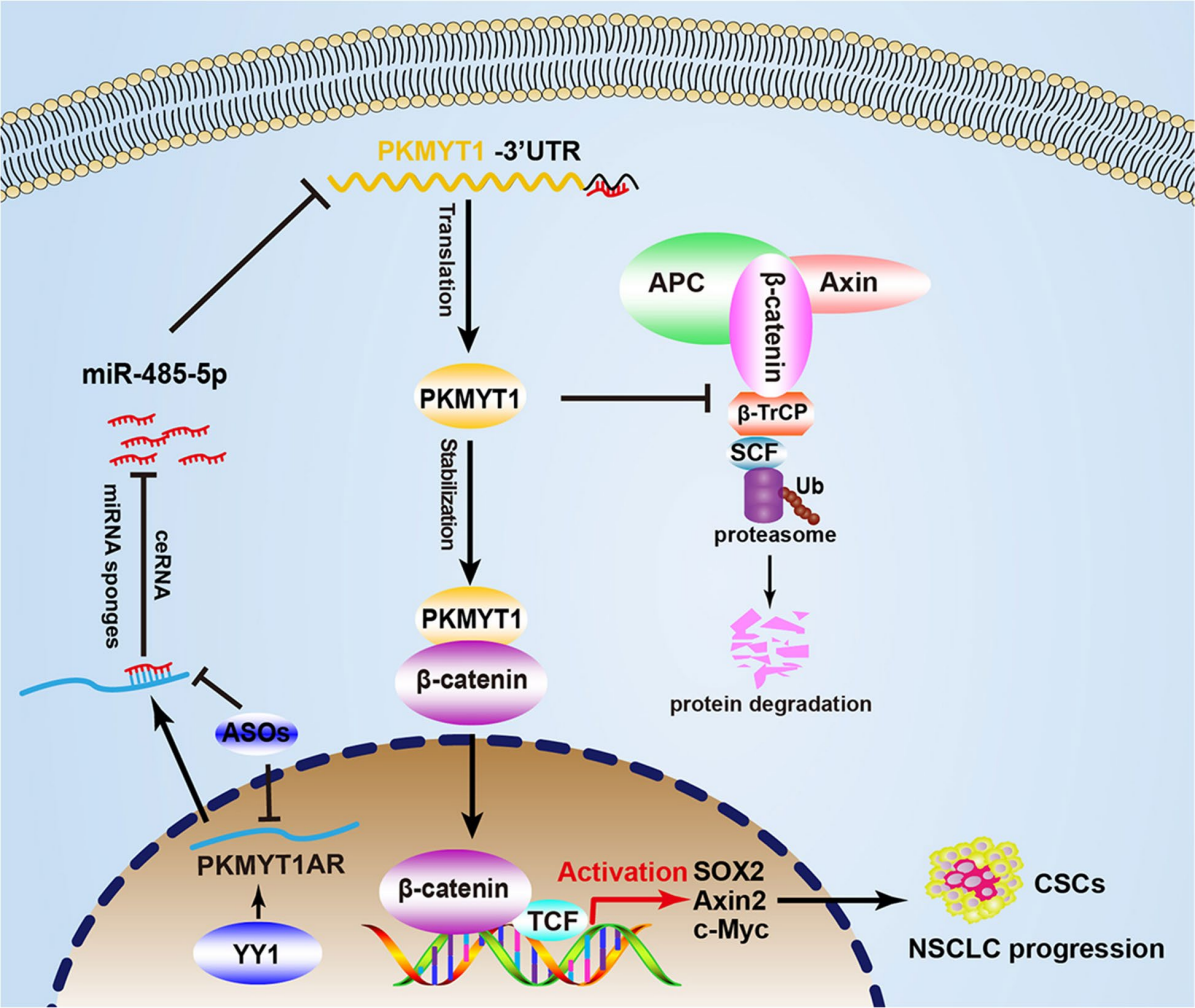
A model demonstrating how PKMYT1AR/miR-485-5p/PKMYT1 axis activates Wnt signaling and cancer stem cell self-renewal ability

To uncover the non-coding RNAs critical for cancer stem cell maintenance, by applying multiple web source available datasets, we were able to identify lncRNA PKMYT1AR as an oncogenic factor promoting NSCLC progression. We found that PKMYT1AR functions as a ceRNA to block the inhibitory effect of miR-485-5p targeting its downstream oncogene PKMYT1 (Fig. 10). Cancer-associated fibroblasts (CAFs) have been demonstrated to promote tumor progression, and miR-485-5p was previously identified to be downregulated in front CAFs from lung adenocarcinoma [42]. Furthermore, miR-485-5p was shown to suppress esophageal squamous cell carcinoma (ESCC) progression by inhibiting flotillin-1 expression leading to reduced EMT process [43], and target PGC-1α to inhibit breast cancer cell metastasis [44]. Here, we demonstrated that PKMYT1AR was increased, whereas miR-485-5p was decreased, in cancerous tissues as well as peripheral blood serum isolated from NSCLC patients compared with reciprocal control groups, suggesting that PKMYT1AR and miR-485-5p functions as an oncogene, and a tumor suppressor, respectively, during NSCLC progression.

As a direct downstream target of miR-485-5p, PKMYT1 was initially identified as a negative regulator of the normal cell cycle transition by inhibiting CDK1-cylin B complex via phosphorylating Tyr14/Tyr15, which is more critical for tumor cell proliferation [45]. Previous studies have also demonstrated that PKMYT1 upregulation promotes the malignancies of glioblastoma, hepatic carcinoma, and colorectal cancer without deciphering the exact molecular mechanisms [13, 15, 46]. Recently, Zhang et al. found that PKMYT1 was increased in HCC and induced tumor cell growth, migration, EMT and metastasis by interacting with and inactivating GSK-3β leading to a constitutive activated Wnt signaling [15], suggesting that PKMYT1 plays potential role regulating cancer stem cells. However, we did not detect significant change on β-catenin phosphorylation sites medicated by GSK-3β, suggesting that the molecular mechanisms mediated by PKMYT1 might be cellular context dependent. Furthermore, we found that PKMYT1 physically interacted with β-catenin proteins blocking the E3-ligase SCF^β-TrCP^ recognition, leading to stabilization of β-catenin proteins and constitutive activation of Wnt signaling, increased self-renewal ability of cancer stem cells in NSCLC (Fig. 10). Given that PKMYT1 protein is localized to the endoplasmic reticulum and Golgi complex except for nuclear, and its catalytic kinase activity was not essential for cell cycle transition [47], our results suggest that the direct protein-protein interaction mediated by PKMYT1 should not be underestimated. For example, PKMYT1 could block other E3 ubiquitin ligase mediated degradation, or promote deubiquitinase mediated stabilization of β-catenin proteins, respectively [48]. Therefore, to decipher the specific role of PKMYT1 in different types of human cancer, indentifying its unique interacting protein complex would be critical.

Stem cells have the capacity to self-renewal and produce differentiated cells, and the decision to divide or differentiate is well controlled by differential molecular events, which, together with the cells generating them, form a niche capable of supporting stem cells. Therefore, in order to improve the clinical outcome, it will be promising to design gene therapy strategy targeting to signaling molecules important for CSCs maintenance, such as PKMYT1AR, miR-485-5p and PKMYT1. Recently, ASO has been applied to inhibit target transcript treating different cancers [41], and we have demonstrated that PKMYT1AR-targeting ASOs are effective both in vitro and in vivo.

## Conclusions

Our findings reveal that PKMYT1AR/miR-485-5p /PKMYT1 axis is important for cancer stem cell maintenance and NSCLC progression both in vitro and in vivo, suggesting that PKMYT1AR, miR-485-5p or PKMYT1 could be used as novel therapeutic targets in the future.

## Supplementary Information

**Additional file 1: Figure S1.** LncRNA PKMYT1AR is highly expressed in NSCLC. **a** The sequence comparison of human-specific lncRNA PKMYT1AR examined by Gentree (http://gentree.ioz.ac.cn/). **b-c** The relative expressions of PKMYT1AR, CD133, SOX2 and CD44 in spheroid- and adherently- cultured A549 and SPC-A1 cells, respectively, examined by Real-time RT-PCR assay. **d** The relative expression level of PKMYT1AR in A549/SPC-A1 parental cells and A549/SPC-A1 DDP resistant cells examined by Real-time RT-PCR. **e** PKMYT1AR was majorly localized in the cytoplasm of SPC-A1 cells using nuclear and cytoplasmic RNA fractionation assay followed by Real-time RT-PCR examination. β-actin and U1 expressions were used as cytoplasmic and nuclear fraction controls, respectively. **f-g** Different forms of full-length lnc-PKMYT1AR were sub-cloned into pcDNA3.1 vector with an C-terminal tagged c-Myc and ATG translational start codon. Anti-myc antibody was used to detect potential expressed proteins. Blue arrowhead indicated NCAPH-myc proteins used as control. **h** The potential transcription factors controlling PKMYT1AR expression were predicted by PROMO. **i** The relative expression level of YY1 in TCGA-LUAD (adenocarcinoma; Normal: 59; Tumor: 533). **j** YY1 high expression correlates with worse overall survival time. **k** Depletion of YY1 reduced PKMYT1AR expression in SPC-A1 cells examined by Real-time RT-PCR. * *P* < 0.05, ** *P* < 0.01, *** *P* < 0.001. **Figure S2.** PKMYT1AR knockdown inhibits tumor cell growth and migration. **a** The cell cycle and epithelial-mesenchymal transition signaling pathways were enriched by GSEA analysis. **b** Establishment of PKMYT1AR overexpression and knockdown cell lines in SPC-A1 verified by Real-time RT-PCR. **c-e** PKMYT1AR knockdown dramatically inhibited SPC-A1 cell proliferation (c) and colony formation abilities (d), (e) is the quantification data for (d). **f** Effect of PKMYT1AR knockdown on the G0/G1 cell cycle transition was tested in SPC-A1 cells by PI staining and flow cytometry. **g** PKMYT1AR knockdown regulated the expressions of cell cycle transition mediators, including CDK2, CDK6, cyclin D1, p21 and p27. Indicated cell extracts were probed with indicated antibodies. **h-i** Knockdown of PKMYT1AR inhibited SPC-A1 cell migration using wound healing (h) and transwell (i) assays. Quantification data were also indicated, and the OD_570_ values for trans-well assay were indicated below. Scale bar=50 μm. **j** Indicated cell extracts were probed with indicated antibodies to examine the expression patterns of cell migration regulators, including E-cadherin, N-cadherin, Vimentin and Slug. **k-l** PKMYT1AR knockdown induced cellular apoptosis in A549 and SPC-A1 cells detected by flow cytometry assay. Quantification data is shown (right). * *P* < 0.05, ** *P* < 0.01, *** *P* < 0.001. **Figure S3.** miR-485-5p inhibits tumor progression. **a** Correlation analysis between PKMYT1AR and miR-216a-5p or miR-6884-5p, respectively, using TCGA-LAUD dataset. **b** The prognostic value of miR-216a-5p and miR-6884-5p in TCGA-LAUD examined by Kaplan-Meier Plotter. **c** The relative expression of PKMYT1AR after over-expression of miR-485-5p, miR-216a-5p and miR-6884-5p in SPC-A1 cells examined by Real-time RT-PCR. **d** The decreased expression of miR-485-5p in GSE74190 dataset. **e** miR-485-5p regulated A549 cell proliferation assay. **f** Relative miR-485-5p expression examined by Real-time RT-PCR assay in indicated cells. **g-h** miR-485-5p regulated SPC-A1 colony formation assay (g), (h) is the quantification data for (g). **i-j** miR-485-5p regulated SPC-A1 cell migration examined by wound healing (i) and trans-well (j) assays. Quantification data were also presented, and the OD_570_ values for trans-well assay were indicated below. Scale bar=50 μm. **k-m** miRNA-485-5p inhibitor overcame PKMYT1AR knockdown effect by cell growth curve (k), colony formation (l) and trans-well (m) assays in A549 cells. Quantification data were also indicated (right). Scale bar=50 μm. **n-p** miRNA-485-5p inhibitor overcame PKMYT1AR knockdown effect by cell growth curve assay (n), colony formation (o) and trans-well (p) assays in SPC-A1 cells. Quantification data were also indicated (right). Scale bar=50 μm. * *P* < 0.05, ** *P* < 0.01, *** *P* < 0.001. **Figure S4.** PKMYT1 is increased in NSCLC. **a** The expression patterns of PKMYT1 in pan-cancer examined by TIMER. **b** The relative expression of PKMYT1 in GSE18842 dataset (Normal: 45; Tumor: 46). **c** PKMYT1 is frequently mutated in various types of human cancer. Mutation (green); Fusion (purple); Amplification (red); Deletion (blue). **d-e** miR-485-5p mimics or inhibitor suppressed or promoted, respectively, PKMYT1 expression in SPC-A1 cells examined by Real-time RT-PCR (d) and immunoblot (e). **f-g** The relative expression level of PKMYT1 in indicated NSCLC cancerous cell lines examined by Real-time RT-PCR (f) and immunoblot (g). **h-i** PKMYT1 proteins were highly expressed in NSCLC cancerous tissues examined by immunoblot (h), (i) is the quantification data for (h). **j** IHC staining of PKMYT1 proteins in normal (non-cancerous lung) tissues (NCLT). **k** The cell cycle and epithelial-mesenchymal transition signaling pathways were enriched by GSEA analysis. * *P* < 0.05, ** *P* < 0.01, *** *P* < 0.001. **Figure S5.** PKMYT1 knockdown inhibits tumor cell proliferation, migration and colony formation abilities. **a-b** Establishment of PKMYT1 knockdown in SPC-A1 cells, verified by Real-time RT-PCR (a) and immunoblot (b). **c** PKMYT1 knockdown dramatically inhibited SPC-A1 cell proliferation using growth curve assay. **d-e** Effect of PKMYT1 knockdown on the G0/G1 cell cycle transition was examined in SPC-A1 cells by PI staining and flow cytometry. (e) is the quantification data for (d). **f** Indicated cell extracts were probed with indicated antibodies. **g-h** PKMYT1 regulated SPC-A1 cell migration examined by wound healing (g) and trans-well (h) assays. Quantification data were also presented, and the OD_570_ values for trans-well assay were indicated below. Scale bar=50 μm. **i** Indicated cell extracts were probed with indicated antibodies to examine the expression patterns of cell migration regulators, including E-cadherin, N-cadherin, Vimentin and Slug. **j** Validation of PKMYT1 over-expression by Real-time RT-PCR (top) and immunoblot (bottom) assays. Blue arrow head: exogenous HA-tagged PKMYT1; black arrow head: endogenous PKMYT1. **k-q** PKMYT1 forced expression overcame PKMYT1AR knockdown effect by cell growth curve (k), wound healing (l-m), trans-well (n-p) and colony formation assays (o-q), (m, p, q) were quantification data for reciprocal assays. **r-s** PKMYT1 knockdown induced cellular apoptosis in A549 and SPC-A1 cells detected by flow cytometry assay. (s) Quantification data for (r). * *P* < 0.05, ** *P* < 0.01, *** *P* < 0.001. **Figure S6.** PKMYT1AR knockdown sensitizes tumor cell response to DDP treatment. **a** The negative correlations between miRNA-485-5p and stem cell maintenance related genes, including the CD44, SOX2, ALDH1 and Nanog, were examined using TCGA-LUAD dataset by Pearson’s correlation analysis. **b-d** PKMYT1AR knockdown promoted DDP induced cellular apoptosis in A549 and SPC-A1 cells detected by SRB assay (b), flow cytometry assay (c) and immunoblot with indicated antibodies (d). (c) is the quantification data for flow cytometry assay. IC_50_ for each cell line was indicated. **e-g** PKMYT1 knockdown promoted DDP induced cellular apoptosis in A549 and SPC-A1 cells detected by SRB assay (e), flow cytometry assay (f) and immunoblot with indicated antibodies (g). (f) is the quantification data for flow cytometry assay. IC_50_ for each cell line was indicated. **h** Schematic picture of xenograft mouse model treated by DDP. Until tumor present around 50 mm^3^, nude mice were injected with DDP every 4 days. **i-k** Representative xenograft tumor images (i), tumor masses (j) and tumor volumes (k) were shown for indicated groups. A549 cells were used. **l-m** Representative IHC staining of Ki67 (l) and CC3 (m) for indicated xenograft tumors. Quantification data is also indicated (right). Scale bar=50 μm. tPARP=total PARP; cPARP=cleaved PARP. * *P* < 0.05, ** *P* < 0.01, *** *P* < 0.001. **Figure S7.** PKMYT1AR inhibition impedes cancer stem cell self-renewal. **a** Relative mRNA expressions of PKMYT1AR and indicated cancer stem cell marker genes in SPC-A1 cells were examined by Real-time RT-PCR upon PKMYT1AR knockdown. **b** Tumor sphere formation abilities of indicated cells after PKMYT1AR knockdown were examined. Scale bar=50 μm. **c** Total extracts of indicated cells were probed with indicated antibodies by immunoblot. **d** Relative mRNA expressions of PKMYT1 and cancer stem cell marker genes in SPC-A1 cells, were examined by Real-time RT-PCR upon PKMYT1 knockdown. **e** Tumor sphere formation abilities of indicated cells after PKMYT1 knockdown were examined. Scale bar=50 μm. **f** Total extracts of indicated cells were probed with indicated antibodies by immunoblot. **g** Relative mRNA expressions of PKMYT1 and cancer stem cell marker genes in SPC-A1 cells, were examined by Real-time RT-PCR with miR-485-5p mimics or miR-NC co-transfection. **h-i** Tumor sphere formation abilities of indicated cells after miR-485-5p mimics or miRNA controls co-expression were examined (h) in SPC-A1 cells. (i) is the quantification data for (h). Scale bar=50 μm. **j** Total extracts of indicated cells were probed with indicated antibodies by immunoblot. **k-l** Rescue effect of PKMYT1AR over-expression on miR-485-5p mimics-mediated phenotype was examined by tumor sphere formation assay in SPC-A1 (k), (l) is the quantification data for (k). Scale bar=50 μm. **m-n** Rescue effect of PKMYT1 over-expression on miR-485-5p mimics-mediated phenotype was examined by tumor sphere formation assay in SPC-A1 (m), (n) is the quantification data for (k). **o-p** Rescue effect of PKMYT1 over-expression on PKMYT1AR depletion-mediated phenotype was examined by tumor sphere formation assay in SPC-A1 (o), (p) is the quantification data for (o). * *P* < 0.05, ** *P* < 0.01, *** *P* < 0.001. **Figure S8.** PKMYT1 stabilizes β-catenin proteins. **a** Wnt signaling pathway activity was examined using TOPFlash reporter assay in indicated cells after Wnt3a or LiCl (100 μM) treatment, respectively. **b** Relative expression of indicated transcripts were examined by Real-time RT-PCR upon PKMYT1 knockdown. **c** Membrane-tethered form of cancer stem cell marker CD133 was examined in indicated cells by flow cytometry assay. Quantification result was indicated (right). **d** Total and phosphorylated forms of β-catenin proteins were examined by immunoblot with indicated antibodies in SPC-A1 cells. **e** Total β-catenin proteins were reduced after PKMYT1 knockdown in SPC-A1 cells, which can be reversed by MG132 (20 μM) treatment. **f** PKMYT1 knockdown inhibited nuclear accumulation of β-catenin proteins in SPC-A1 cells by immunoblot. **g-h** Total extracts of indicated cells treated by cycloheximide (CHX: 100 μg/mL) were examined by immunoblot with indicated antibodies. (h) is the quantification data for (g). **i** Co-IP assay to detect the endogenous protein interactions among β-catenin, PKMYT1 and βTrCP1 in A549 cells. **j-l** Co-IP assay to detect the exogenous protein interactions among β-catenin, PKMYT1 and β-TrCP1 in HEK-293 T cell. β-cat-Flag=β-catenin-Flag. * *P* < 0.05, ** *P* < 0.01, *** *P* < 0.001. **Figure S9.** PKMYT1AR targeting ASOs inhibit tumor progression. **a** The relative expressions of PKMYT1AR transcripts were examined by Real-time RT-PCR after indicated ASOs transfection. **b** Knockdown of PKMYT1AR inhibited cell proliferation examined by growth curve assays. **c-d** PKMYT1AR targeting ASOs repressed cell growth by colony formation assay. (d) is the quantification data for (c). **e-f** PKMYT1AR targeting ASOs inhibited cell migration by trans-well assay (e). (f) is the quantification data for (e). Scale bar=50 μm. **g-h** PKMYT1AR targeting ASOs inhibited cancer stem cell self-renewal ability by tumor-sphere formation assay (g). (h) is the quantification data for (g). **i** PKMYT1AR targeting ASOs inhibited cancer stem cell marker genes expressions examined by immunoblot with indicated antibodies. **j-k** Representative IHC staining of CD44, Sox2 and β-catenin for indicated xenograft tumors (j). (k) is the quantification data for (j). Scale bar=50 μm. * *P* < 0.05, ** *P* < 0.01, *** *P* < 0.001. **Table S1.** The candidate lncRNAs were identified by integrative analyses using various GEO datasets. **Table S2.** Antibodies and oligos used in this study. **Table S3.** Top 50% predicted downstream target genes of miRNA-485-5p examined by StarBase, Targetscan and miRWalk, respectively. **Table S4.** The full names of cancer types shown in Fig. S4a. **Table S5.** The dataset used in the Fig. S4c. **Table S6.** The pathological characteristics of patients with non-small cell lung cancer (NSCLC), and health donors.

## Data Availability

All data that support the findings of this study are available from the corresponding authors upon reasonable request.

## Abbreviations

NSCLC: Non-small cell lung cancer
PKMYT1AR: PKMYT1 associated lncRNA
PKMYT1: Protein kinase, membrane-associated tyrosine/threonine 1
LUAD: Lung adenocarcinoma
LUSC: Lung squamous cell carcinoma
ceRNA: Competing endogenous RNA
LncRNA: Long non-coding RNA
WT: Wild-type
DDP: Cisplatinum
ROC: Receiver operating characteristic
AUC: Area under the curve
NCLT: Non-cancerous lung tissue
ASO: Antisense oligonucleotide
3’-UTR: 3’-untranslated region
OS: Overall survival
RNAi: RNA interference
CSCs: Cancer stem cells
qRT-PCR: Quantitative reverse transcription polymerase chain reaction;
FISH: Fluorescence in situ hybridization
EMT: Epithelial-mesenchymal transition
miRNA: MicroRNA
shRNA: Short hairpin RNAs
IHC: Immunohistochemistry
FBS: Fetal calf serum
GEO: Gene Expression Omnibus
TCGA: The Cancer Genome Atlas
CC3: Cleaved Caspase3
DAPI: 4,6-diamidino-2-phenylindole
CHX: Cycloheximide
PBS: Phosphate-buffered saline
