# Supplementary material for: LncRNA PKMYT1AR promotes cancer stem cell maintenance in non-small cell lung cancer via activating Wnt signaling pathway
Source: Mol Cancer. 2021 Dec 2;20:156. doi: 10.1186/s12943-021-01469-6 (PMC8638142; doi:10.1186/s12943-021-01469-6)
Supplement: Supplementary file 1 — Additional file 1: Figure S1. LncRNA PKMYT1AR is highly expressed in NSCLC. a The sequence comparison of human-specific lncRNA PKMYT1AR examined by Gentree (http://gentree.ioz.ac.cn/). b-c The relative expressions of PKMYT1AR, CD133, SOX2 and CD44 in spheroid- and adherently- cultured A549 and SPC-A1 cells, respectively, examined by Real-time RT-PCR assay. d The relative expression level of PKMYT1AR in A549/SPC-A1 parental cells and A549/SPC-A1 DDP resistant cells examined by Real-time RT-PCR. e PKMYT1AR was majorly localized in the cytoplasm of SPC-A1 cells using nuclear and cytoplasmic RNA fractionation assay followed by Real-time RT-PCR examination. β-actin and U1 expressions were used as cytoplasmic and nuclear fraction controls, respectively. f-g Different forms of full-length lnc-PKMYT1AR were sub-cloned into pcDNA3.1 vector with an C-terminal tagged c-Myc and ATG translational start codon. Anti-myc antibody was used to detect potential expressed proteins. Blue arrowhead indicated NCAPH-myc proteins used as control. h The potential transcription factors controlling PKMYT1AR expression were predicted by PROMO. i The relative expression level of YY1 in TCGA-LUAD (adenocarcinoma; Normal: 59; Tumor: 533). j YY1 high expression correlates with worse overall survival time. k Depletion of YY1 reduced PKMYT1AR expression in SPC-A1 cells examined by Real-time RT-PCR. * P < 0.05, ** P < 0.01, *** P < 0.001. Figure S2. PKMYT1AR knockdown inhibits tumor cell growth and migration. a The cell cycle and epithelial-mesenchymal transition signaling pathways were enriched by GSEA analysis. b Establishment of PKMYT1AR overexpression and knockdown cell lines in SPC-A1 verified by Real-time RT-PCR. c-e PKMYT1AR knockdown dramatically inhibited SPC-A1 cell proliferation (c) and colony formation abilities (d), (e) is the quantification data for (d). f Effect of PKMYT1AR knockdown on the G0/G1 cell cycle transition was tested in SPC-A1 cells by PI staining and flow cytometry. g PKMYT [file 12943_2021_1469_MOESM1_ESM.doc]

**LncRNA PKMYT1AR promotes cancer stem cell maintenance in non-small cell lung cancer via activating Wnt signaling pathway**

Yaomei He^1,2 ‡^, Xiulin Jiang^1,2 ‡^, Lincan Duan^3, ‡^, Qiuxia Xiong^4^, Yixiao Yuan^3^, Peishen Liu^1,2^, Liping Jiang^1^, Qiushuo Shen^5^, Song Zhao^5^, Cuiping Yang^1,*^, Yongbin Chen^1,6, *^

1. Key Laboratory of Animal Models and Human Disease Mechanisms of Chinese Academy of Sciences & Yunnan Province, Kunming Institute of Zoology, Kunming, Yunnan 650223, China.
2. Kunming College of Life Science, University of Chinese Academy of Sciences, Beijing, 100049, China.
3. Department of Thoracic Surgery, the Third Affiliated Hospital of Kunming Medical University, Kunming, Yunnan 650118, China.
4. Department of Clinical Laboratory, the First Affiliated Hospital of Kunming Medical University, Kunming, 650032, China.
5. Department of Thoracic Surgery, the First Affiliated Hospital of Zhengzhou University, Zhengzhou 450052, China.
6. Center for Excellence in Animal Evolution and Genetics, Chinese Academy of Sciences, Kunming, Yunnan 650223, China.

‡ These authors contributed equally to this work.

^*^ Correspondence email: Yongbin Chen: [ybchen@mail.kiz.ac.cn](mailto:ybchen@mail.kiz.ac.cn)

Cuiping Yang : [cuipingyang@mail.kiz.ac.cn](mailto:cuipingyang@mail.kiz.ac.cn)

**Supplementary Information**

**
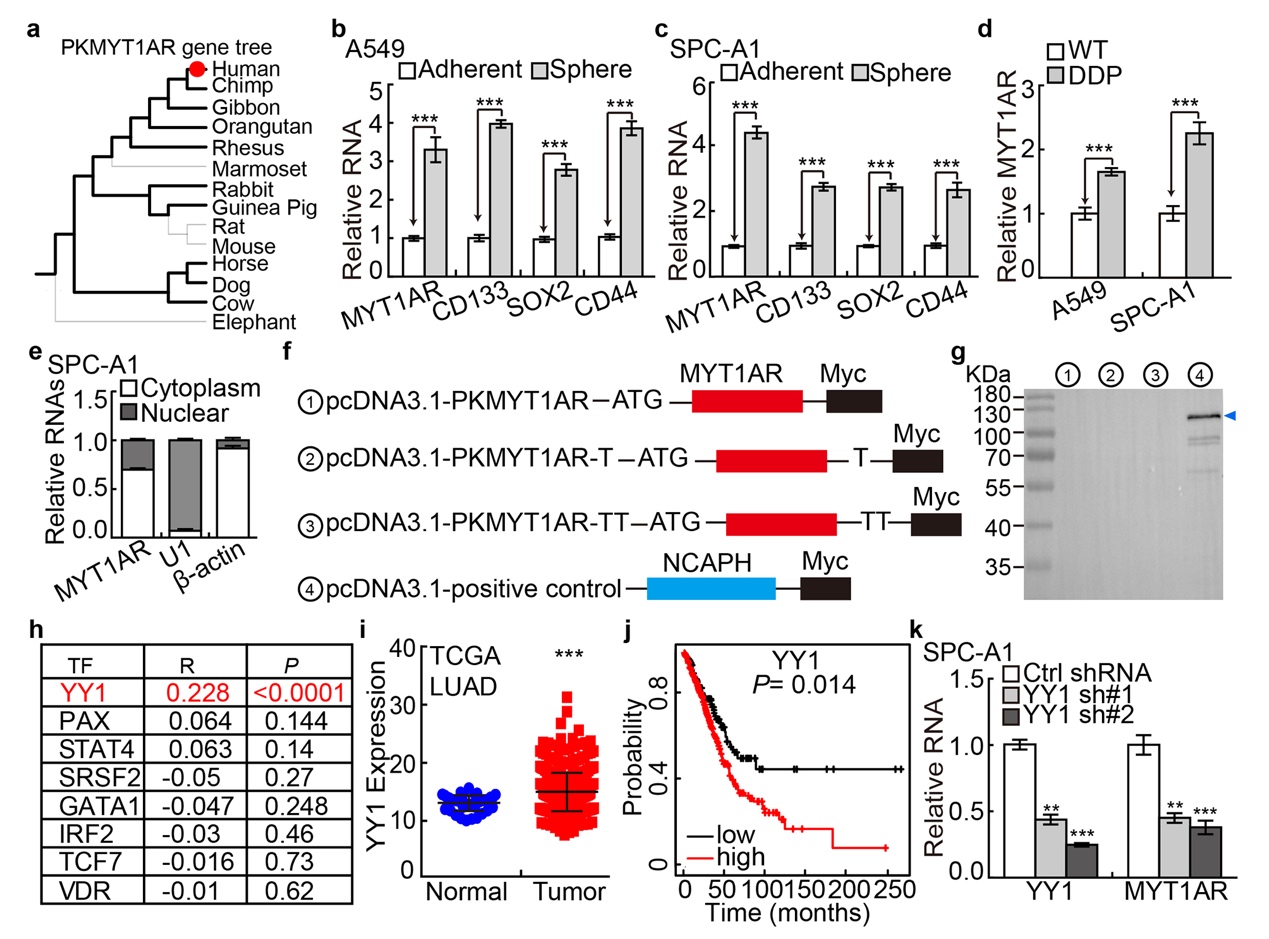
**

**Fig. S1 LncRNA PKMYT1AR is highly expressed in NSCLC.** **a** The sequence comparison of human-specific lncRNA PKMYT1AR examined by Gentree (<http://gentree.ioz.ac.cn/>). **b-c** The relative expressions of PKMYT1AR, CD133, SOX2 and CD44 in spheroid- and adherently- cultured A549 and SPC-A1 cells, respectively, examined by Real-time RT-PCR assay. **d** The relative expression level of PKMYT1AR in A549/SPC-A1 parental cells and A549/SPC-A1 DDP resistant cells examined by Real-time RT-PCR. **e** PKMYT1AR was majorly localized in the cytoplasm of SPC-A1 cells using nuclear and cytoplasmic RNA fractionation assay followed by Real-time RT-PCR examination. β-actin and U1 expressions were used as cytoplasmic and nuclear fraction controls, respectively. **f-g** Different forms of full-length lnc-PKMYT1AR were sub-cloned into pcDNA3.1 vector with an C-terminal tagged c-Myc and ATG translational start codon. Anti-myc antibody was used to detect potential expressed proteins. Blue arrowhead indicated NCAPH-myc proteins used as control. **h** The potential transcription factors controlling PKMYT1AR expression were predicted by PROMO. **i** The relative expression level of YY1 in TCGA-LUAD (adenocarcinoma; Normal: 59; Tumor: 533). **j** YY1 high expression correlates with worse overall survival time. **k** Depletion of YY1 reduced PKMYT1AR expression in SPC-A1 cells examined by Real-time RT-PCR. * *P* < 0.05, ** *P* < 0.01, *** *P* < 0.001.


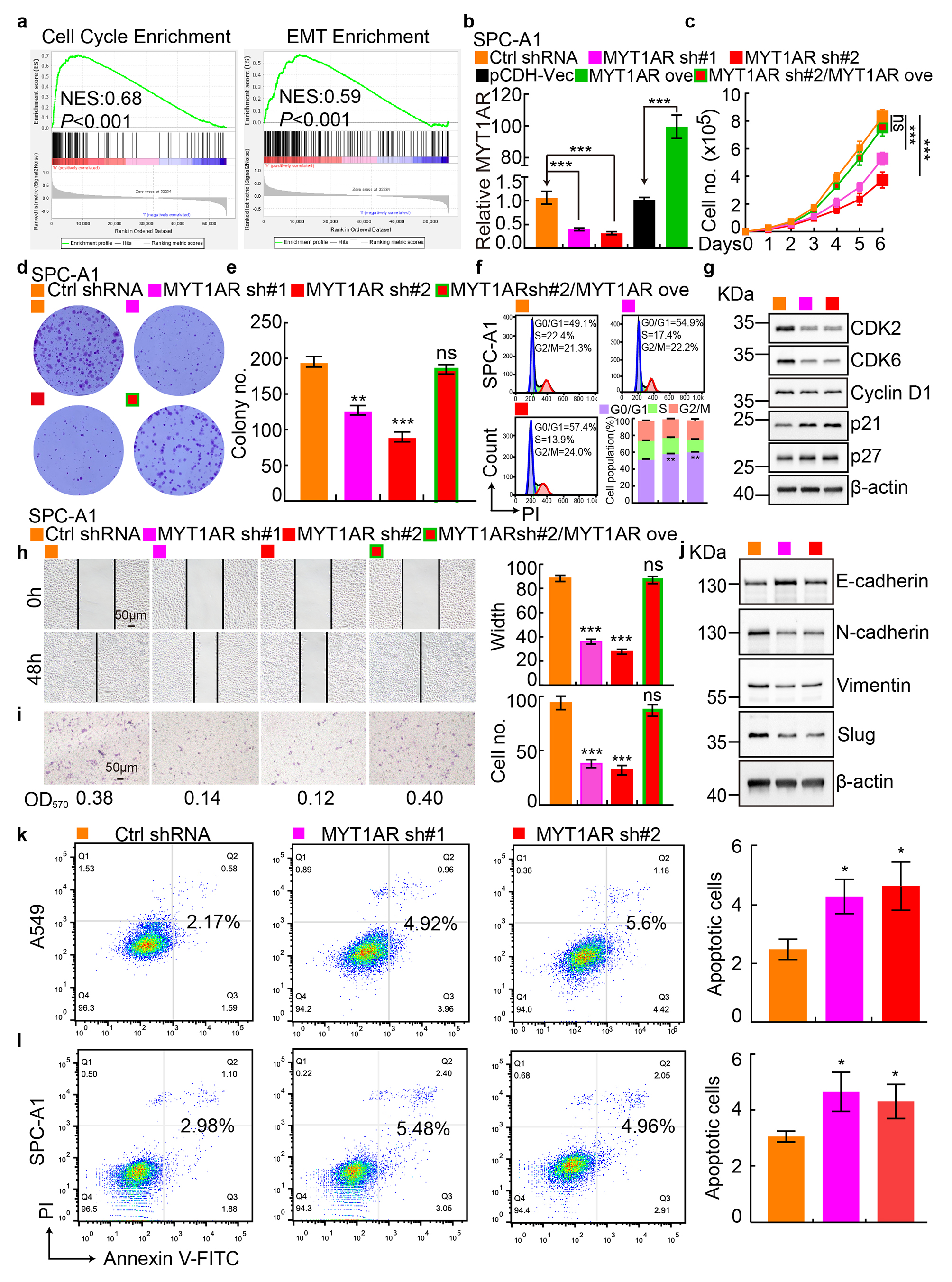


**Fig. S2 PKMYT1AR knockdown inhibits tumor cell growth and migration. a** The cell cycle and epithelial-mesenchymal transition signaling pathways were enriched by GSEA analysis. **b** Establishment of PKMYT1AR overexpression and knockdown cell lines in SPC-A1 verified by Real-time RT-PCR. **c-e** PKMYT1AR knockdown dramatically inhibited SPC-A1 cell proliferation (c) and colony formation abilities (d), (e) is the quantification data for (d). **f** Effect of PKMYT1AR knockdown on the G0/G1 cell cycle transition was tested in SPC-A1 cells by PI staining and flow cytometry. **g** PKMYT1AR knockdown regulated the expressions of cell cycle transition mediators, including CDK2, CDK6, cyclin D1, p21 and p27. Indicated cell extracts were probed with indicated antibodies. **h-i** Knockdown of PKMYT1AR inhibited SPC-A1 cell migration using wound healing (h) and transwell (i) assays. Quantification data were also indicated, and the OD_570_ values for trans-well assay were indicated below. Scale bar=50 μm. **j** Indicated cell extracts were probed with indicated antibodies to examine the expression patterns of cell migration regulators, including E-cadherin, N-cadherin, Vimentin and Slug. **k-l** PKMYT1AR knockdown induced cellular apoptosis in A549 and SPC-A1 cells detected by flow cytometry assay. Quantification data is shown (right). * *P* < 0.05, ** *P* < 0.01, *** *P* < 0.001.


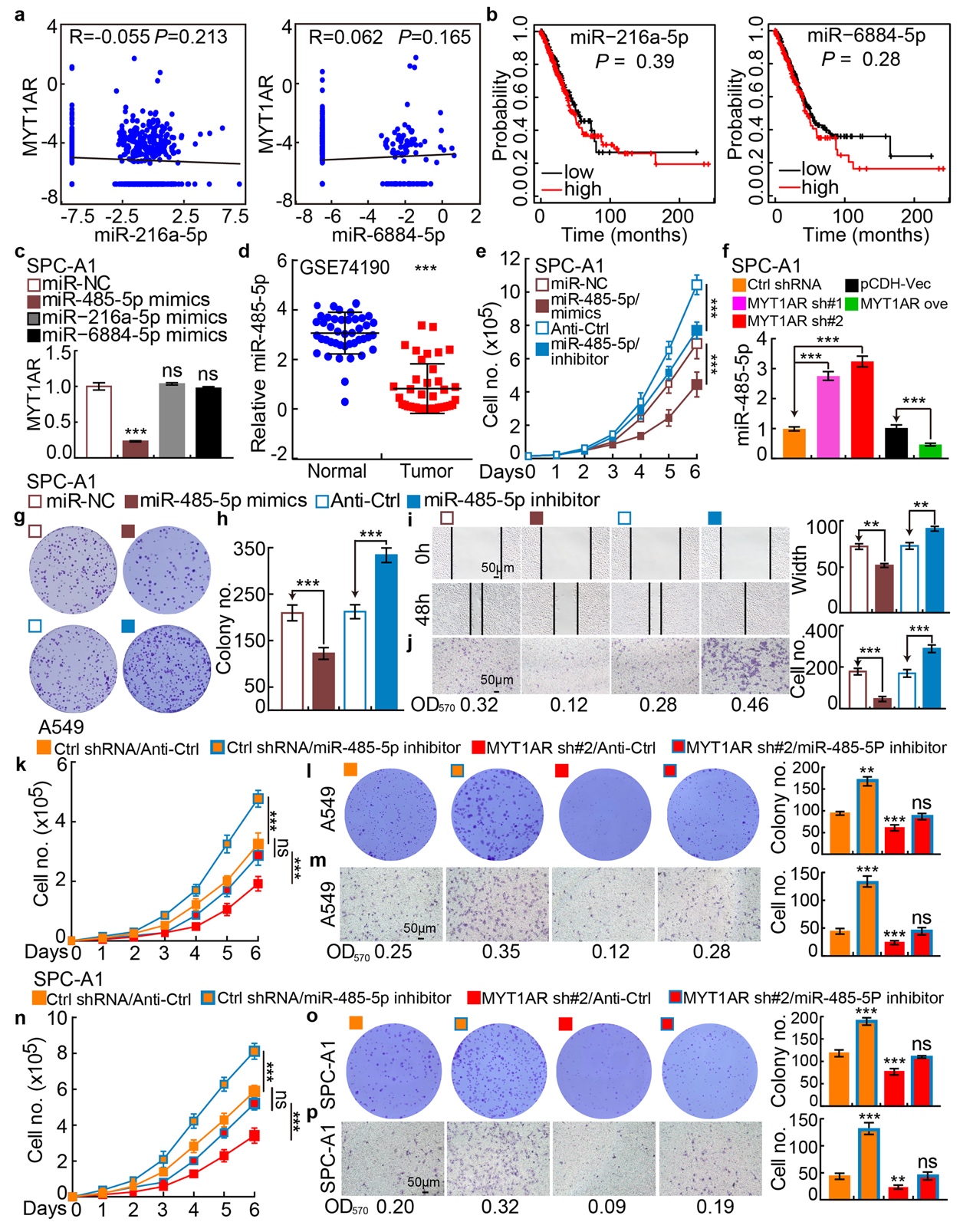


**Fig. S3** **miR-485-5p inhibits tumor progression. a** Correlation analysis between PKMYT1AR and miR-216a-5p or miR-6884-5p, respectively, using TCGA-LAUD dataset. **b** The prognostic value of miR-216a-5p and miR-6884-5p in TCGA-LAUD examined by Kaplan-Meier Plotter. **c** The relative expression of PKMYT1AR after over-expression of miR-485-5p, miR-216a-5p and miR-6884-5p in SPC-A1 cells examined by Real-time RT-PCR. **d** The decreased expression of miR-485-5p in GSE74190 dataset. **e** miR-485-5p regulated A549 cell proliferation assay. **f** Relative miR-485-5p expression examined by Real-time RT-PCR assay in indicated cells. **g-h** miR-485-5p regulated SPC-A1 colony formation assay (g), (h) is the quantification data for (g). **i-j** miR-485-5p regulated SPC-A1 cell migration examined by wound healing (i) and trans-well (j) assays. Quantification data were also presented, and the OD_570_ values for trans-well assay were indicated below. Scale bar=50 μm. **k-m** miRNA-485-5p inhibitor overcame PKMYT1AR knockdown effect by cell growth curve (k), colony formation (l) and trans-well (m) assays in A549 cells. Quantification data were also indicated (right). Scale bar=50 μm. **n-p** miRNA-485-5p inhibitor overcame PKMYT1AR knockdown effect by cell growth curve assay (n), colony formation (o) and trans-well (p) assays in SPC-A1 cells. Quantification data were also indicated (right). Scale bar=50 μm. * *P* < 0.05, ** *P* < 0.01, *** *P* < 0.001.


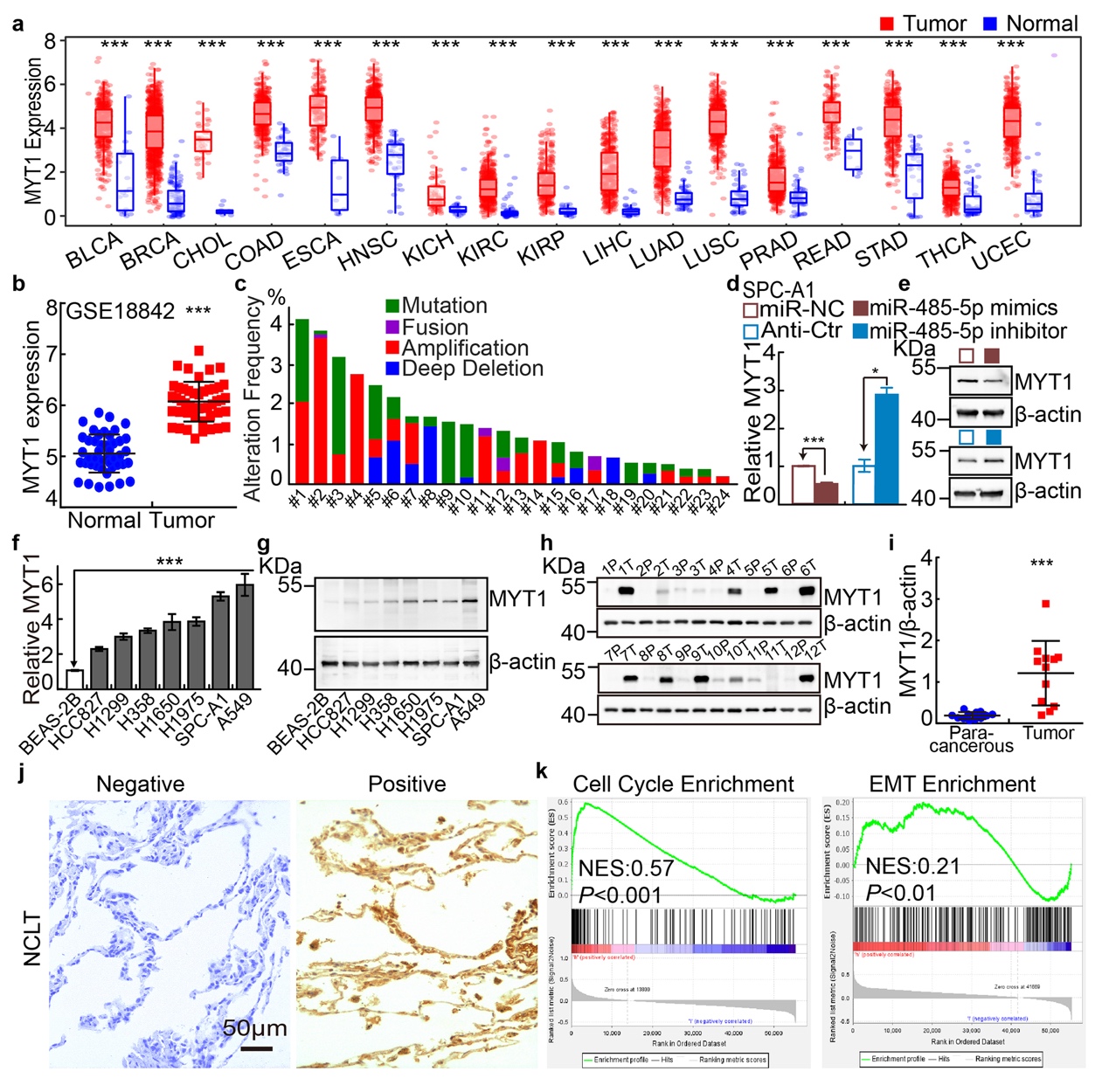


**Fig. S4 PKMYT1 is increased in NSCLC. a** The expression patterns of PKMYT1 in pan-cancer examined by TIMER. **b** The relative expression of PKMYT1 in GSE18842 dataset (Normal: 45; Tumor: 46). **c** PKMYT1 is frequently mutated in various types of human cancer. Mutation (green); Fusion (purple); Amplification (red); Deletion (blue). **d-e** miR-485-5p mimics or inhibitor suppressed or promoted, respectively, PKMYT1 expression in SPC-A1 cells examined by Real-time RT-PCR (d) and immunoblot (e). **f-g** The relative expression level of PKMYT1 in indicated NSCLC cancerous cell lines examined by Real-time RT-PCR (f) and immunoblot (g). **h-i** PKMYT1 proteins were highly expressed in NSCLC cancerous tissues examined by immunoblot (h), (i) is the quantification data for (h). **j** IHC staining of PKMYT1 proteins in normal (non-cancerous lung) tissues (NCLT). **k** The cell cycle and epithelial-mesenchymal transition signaling pathways were enriched by GSEA analysis. * *P* < 0.05, ** *P* < 0.01, *** *P* < 0.001.


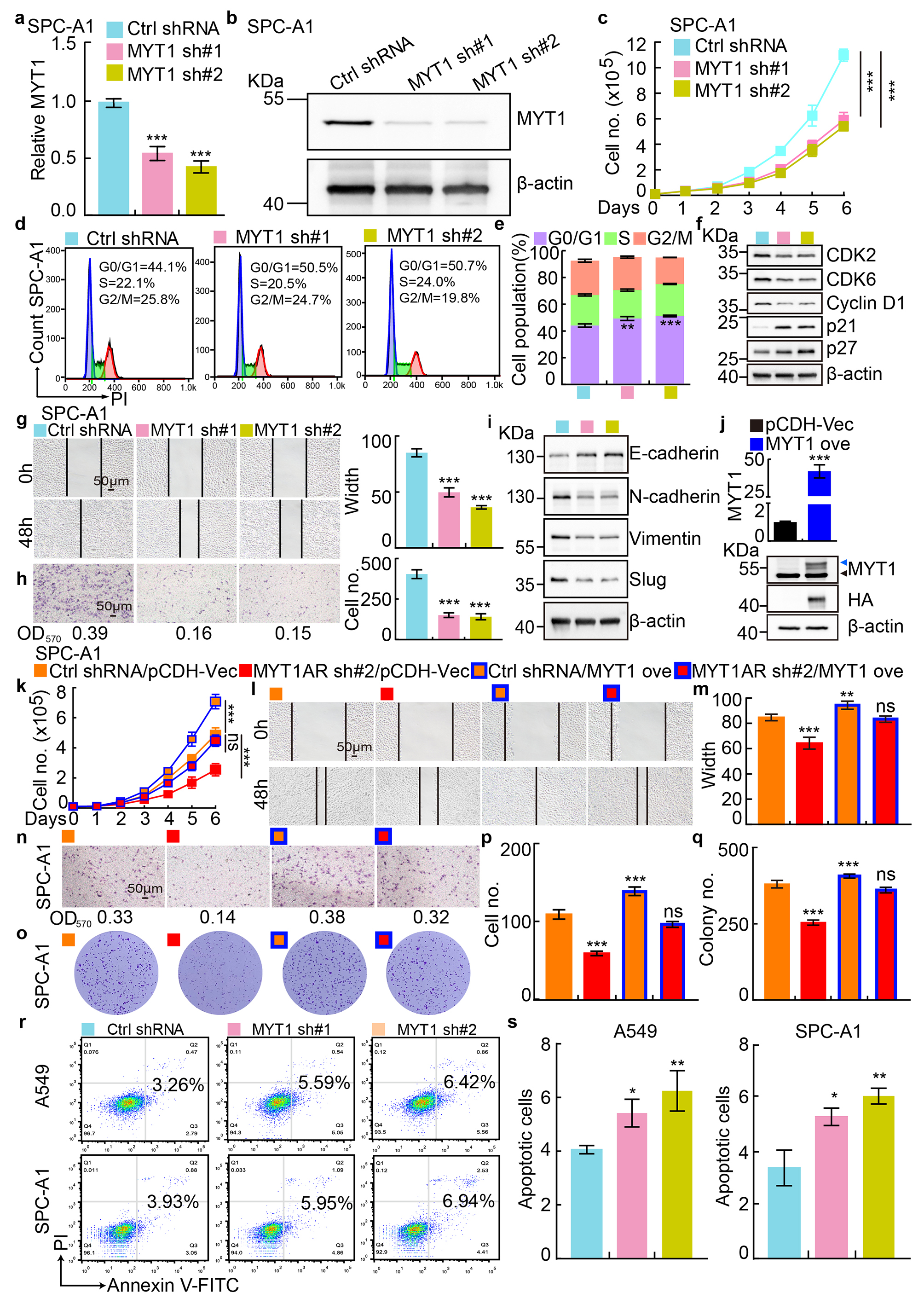


**Fig. S5 PKMYT1 knockdown inhibits tumor cell proliferation, migration and colony formation abilities. a-b** Establishment of PKMYT1 knockdown in SPC-A1 cells, verified by Real-time RT-PCR (a) and immunoblot (b). **c** PKMYT1 knockdown dramatically inhibited SPC-A1 cell proliferation using growth curve assay. **d-e** Effect of PKMYT1 knockdown on the G0/G1 cell cycle transition was examined in SPC-A1 cells by PI staining and flow cytometry. (e) is the quantification data for (d). **f** Indicated cell extracts were probed with indicated antibodies. **g-h** PKMYT1 regulated SPC-A1 cell migration examined by wound healing (g) and trans-well (h) assays. Quantification data were also presented, and the OD_570_ values for trans-well assay were indicated below. Scale bar=50 μm. **i** Indicated cell extracts were probed with indicated antibodies to examine the expression patterns of cell migration regulators, including E-cadherin, N-cadherin, Vimentin and Slug. **j** Validation of PKMYT1 over-expression by Real-time RT-PCR (top) and immunoblot (bottom) assays. Blue arrow head: exogenous HA-tagged PKMYT1; black arrow head: endogenous PKMYT1. **k-q** PKMYT1 forced expression overcame PKMYT1AR knockdown effect by cell growth curve (k), wound healing (l-m), trans-well (n-p) and colony formation assays (o-q), (m, p, q) were quantification data for reciprocal assays. **r-s** PKMYT1 knockdown induced cellular apoptosis in A549 and SPC-A1 cells detected by flow cytometry assay. (s) Quantification data for (r). * *P* < 0.05, ** *P* < 0.01, *** *P* < 0.001.


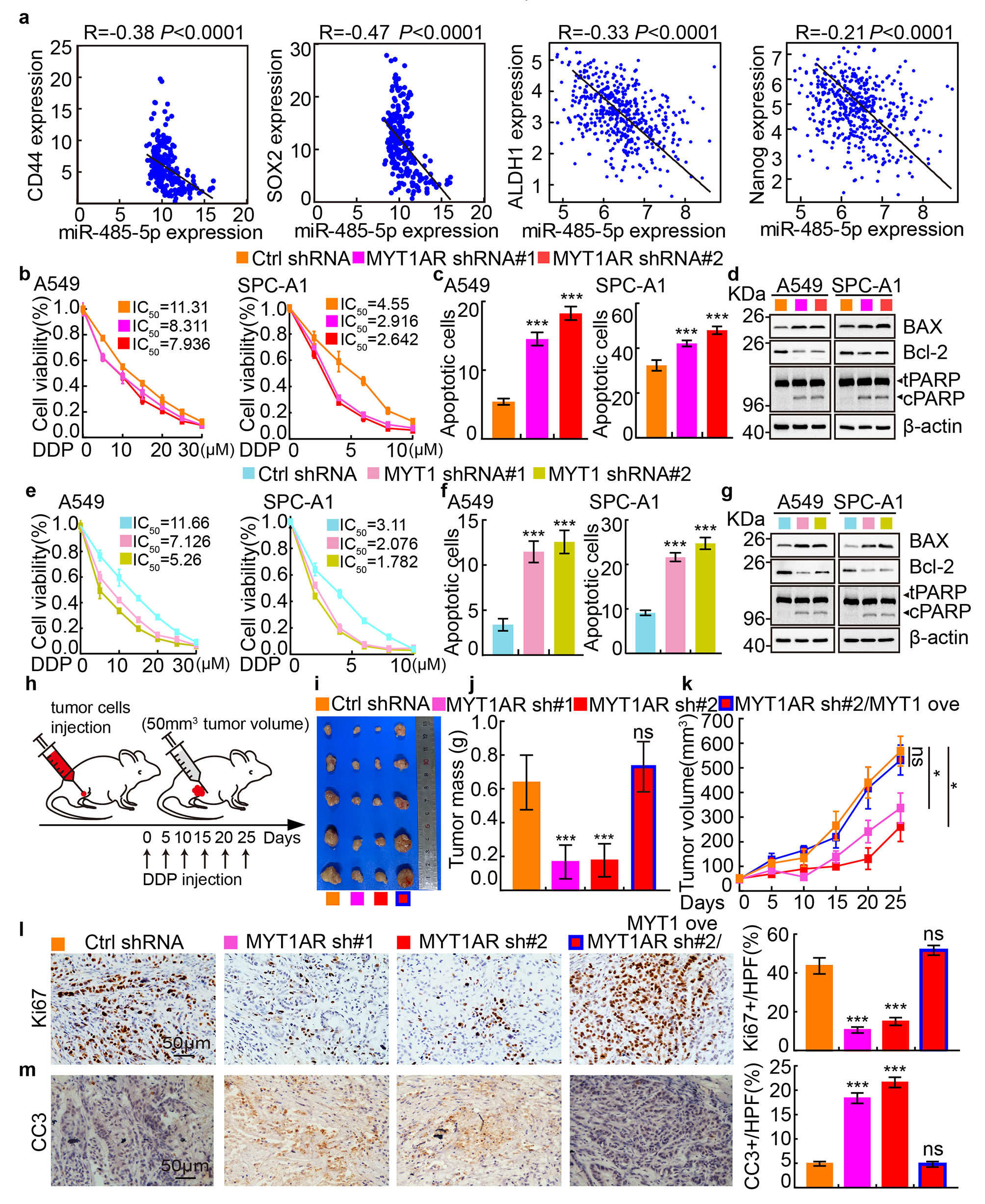


**Fig. S6 PKMYT1AR knockdown sensitizes tumor cell response to DDP treatment. a** The negative correlations between miRNA-485-5p and stem cell maintenance related genes, including the CD44, SOX2, ALDH1 and Nanog, were examined using TCGA-LUAD dataset by Pearson’s correlation analysis. **b-d** PKMYT1AR knockdown promoted DDP induced cellular apoptosis in A549 and SPC-A1 cells detected by SRB assay (b), flow cytometry assay (c) and immunoblot with indicated antibodies (d). (c) is the quantification data for flow cytometry assay. IC_50_ for each cell line was indicated. **e-g** PKMYT1 knockdown promoted DDP induced cellular apoptosis in A549 and SPC-A1 cells detected by SRB assay (e), flow cytometry assay (f) and immunoblot with indicated antibodies (g). (f) is the quantification data for flow cytometry assay. IC_50_ for each cell line was indicated. **h** Schematic picture of xenograft mouse model treated by DDP. Until tumor present around 50 mm^3^, nude mice were injected with DDP every 4 days. **i-k** Representative xenograft tumor images (i), tumor masses (j) and tumor volumes (k) were shown for indicated groups. A549 cells were used. **l-m** Representative IHC staining of Ki67 (l) and CC3 (m) for indicated xenograft tumors. Quantification data is also indicated (right). Scale bar=50 μm. tPARP=total PARP; cPARP=cleaved PARP. * *P* < 0.05, ** *P* < 0.01, *** *P* < 0.001.


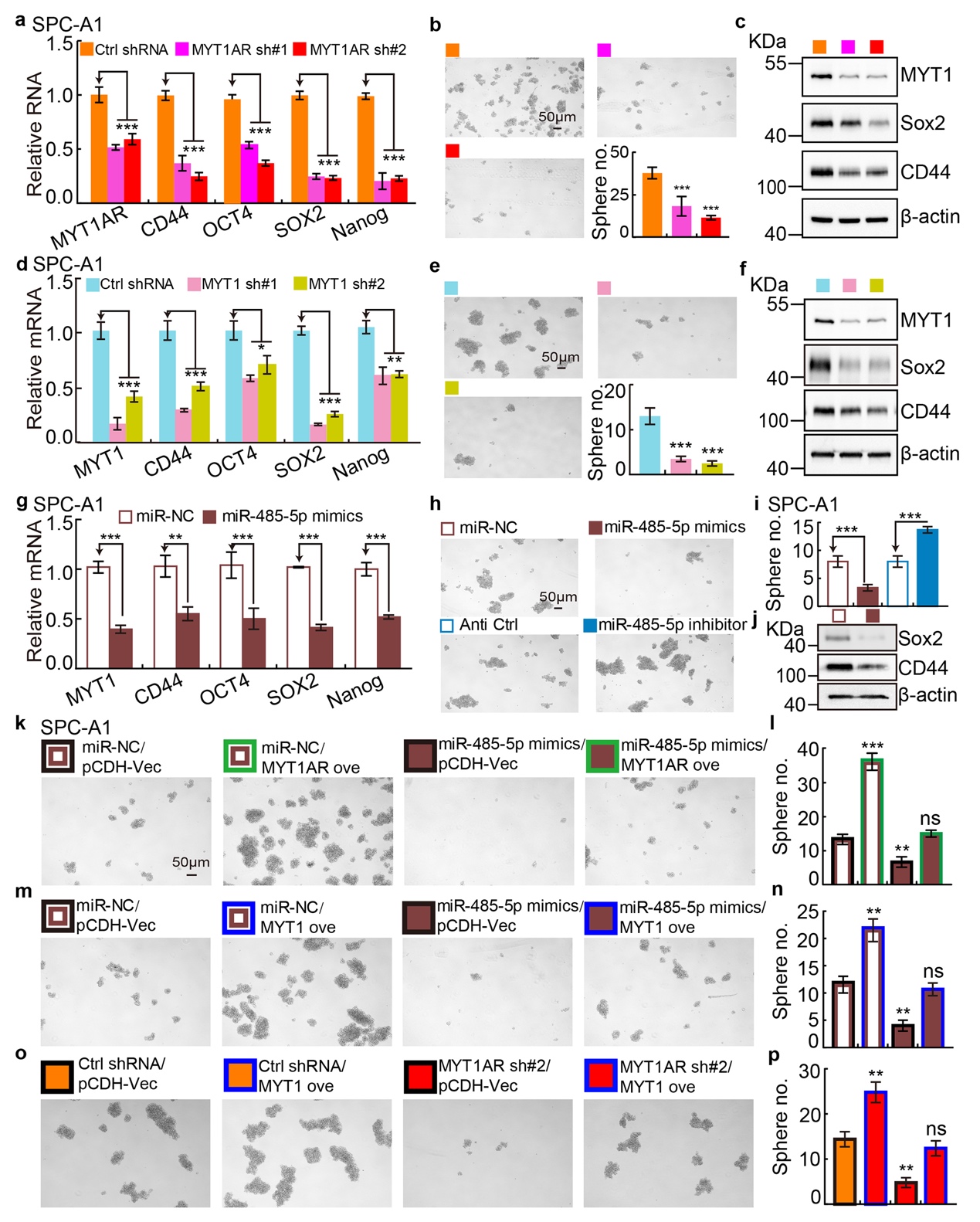


**Fig. S7 PKMYT1AR inhibition impedes cancer stem cell self-renewal. a** Relative mRNA expressions of PKMYT1AR and indicated cancer stem cell marker genes in SPC-A1 cells were examined by Real-time RT-PCR upon PKMYT1AR knockdown. **b** Tumor sphere formation abilities of indicated cells after PKMYT1AR knockdown were examined. Scale bar=50 μm. **c** Total extracts of indicated cells were probed with indicated antibodies by immunoblot. **d** Relative mRNA expressions of PKMYT1 and cancer stem cell marker genes in SPC-A1 cells, were examined by Real-time RT-PCR upon PKMYT1 knockdown. **e** Tumor sphere formation abilities of indicated cells after PKMYT1 knockdown were examined. Scale bar=50 μm. **f** Total extracts of indicated cells were probed with indicated antibodies by immunoblot. **g** Relative mRNA expressions of PKMYT1 and cancer stem cell marker genes in SPC-A1 cells, were examined by Real-time RT-PCR with miR-485-5p mimics or miR-NC co-transfection. **h-i** Tumor sphere formation abilities of indicated cells after miR-485-5p mimics or miRNA controls co-expression were examined (h) in SPC-A1 cells. (i) is the quantification data for (h). Scale bar=50 μm. **j** Total extracts of indicated cells were probed with indicated antibodies by immunoblot. **k-l** Rescue effect of PKMYT1AR over-expression on miR-485-5p mimics-mediated phenotype was examined by tumor sphere formation assay in SPC-A1 (k), (l) is the quantification data for (k). Scale bar=50 μm. **m-n** Rescue effect of PKMYT1 over-expression on miR-485-5p mimics-mediated phenotype was examined by tumor sphere formation assay in SPC-A1 (m), (n) is the quantification data for (k). **o-p** Rescue effect of PKMYT1 over-expression on PKMYT1AR depletion-mediated phenotype was examined by tumor sphere formation assay in SPC-A1 (o), (p) is the quantification data for (o). * *P* < 0.05, ** *P* < 0.01, *** *P* < 0.001.


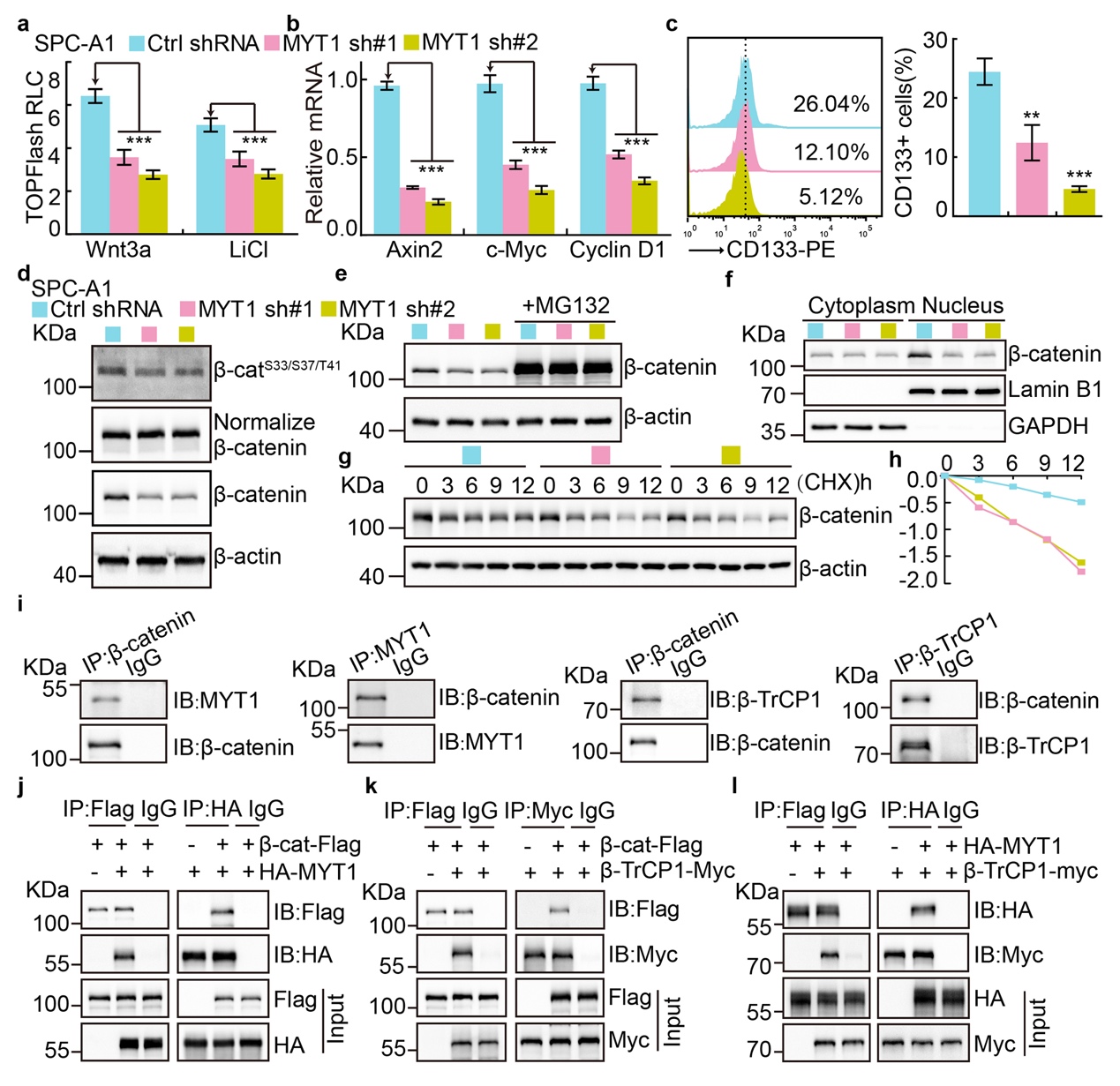


**Fig. S8 PKMYT1 stabilizes β-catenin proteins. a** Wnt signaling pathway activity was examined using TOPFlash reporter assay in indicated cells after Wnt3a or LiCl (100 μM) treatment, respectively. **b** Relative expression of indicated transcripts were examined by Real-time RT-PCR upon PKMYT1 knockdown. **c** Membrane-tethered form of cancer stem cell marker CD133 was examined in indicated cells by flow cytometry assay. Quantification result was indicated (right). **d** Total and phosphorylated forms of β-catenin proteins were examined by immunoblot with indicated antibodies in SPC-A1 cells. **e** Total β-catenin proteins were reduced after PKMYT1 knockdown in SPC-A1 cells, which can be reversed by MG132 (20 μM) treatment. **f** PKMYT1 knockdown inhibited nuclear accumulation of β-catenin proteins in SPC-A1 cells by immunoblot. **g-h** Total extracts of indicated cells treated by cycloheximide (CHX: 100 μg/mL) were examined by immunoblot with indicated antibodies. (h) is the quantification data for (g). **i** Co-IP assay to detect the endogenous protein interactions among β-catenin, PKMYT1 and βTrCP1 in A549 cells. **j-l** Co-IP assay to detect the exogenous protein interactions among β-catenin, PKMYT1 and β-TrCP1 in HEK-293 T cell. β-cat-Flag=β-catenin-Flag. * *P* < 0.05, ** *P* < 0.01, *** *P* < 0.001.

**
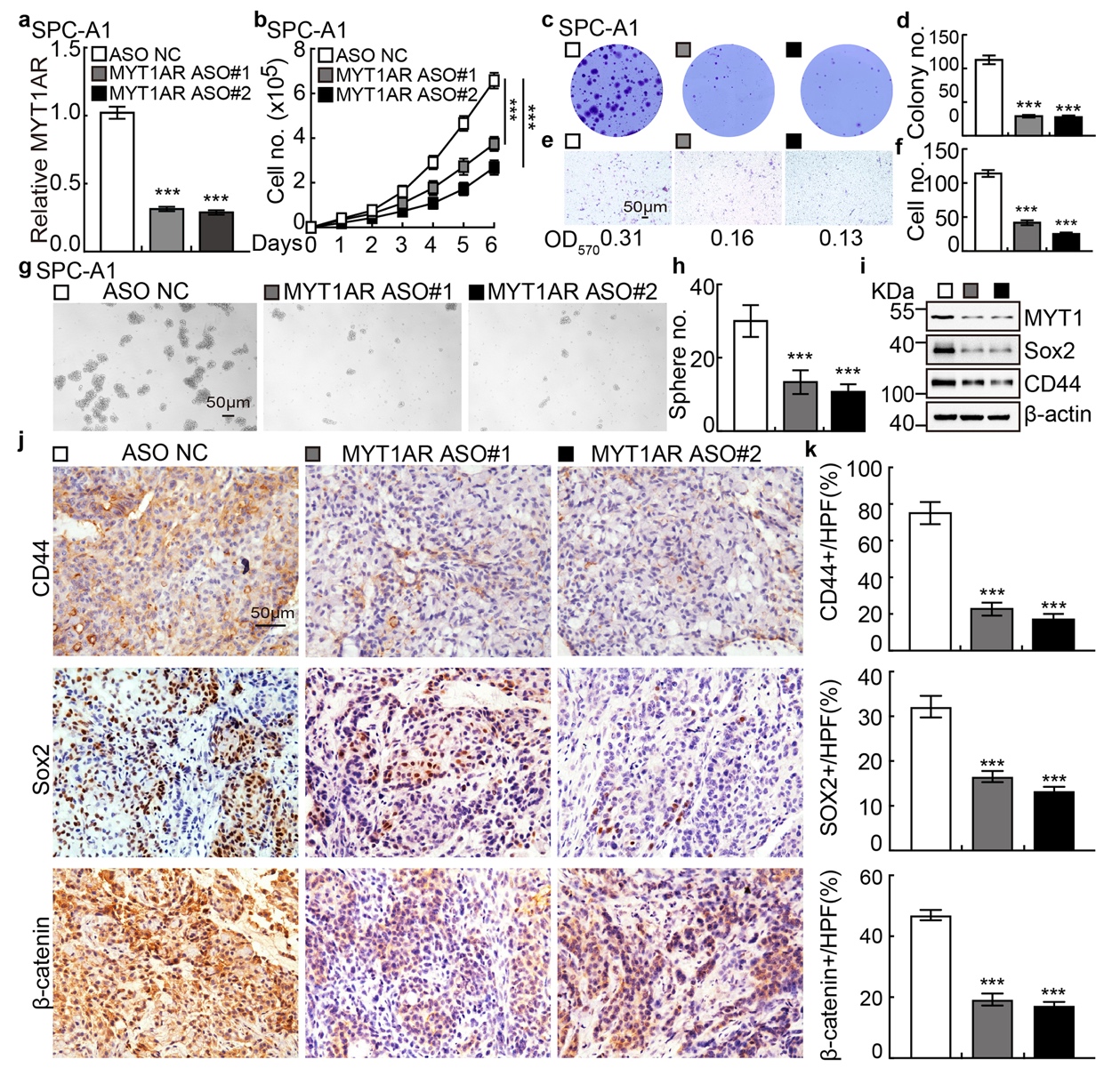
**

**Fig. S9 PKMYT1AR targeting ASOs inhibit tumor progression. a** The relative expressions of PKMYT1AR transcripts were examined by Real-time RT-PCR after indicated ASOs transfection. **b** Knockdown of PKMYT1AR inhibited cell proliferation examined by growth curve assays. **c-d** PKMYT1AR targeting ASOs repressed cell growth by colony formation assay. (d) is the quantification data for (c). **e-f** PKMYT1AR targeting ASOs inhibited cell migration by trans-well assay (e). (f) is the quantification data for (e). Scale bar=50 μm. **g-h** PKMYT1AR targeting ASOs inhibited cancer stem cell self-renewal ability by tumor-sphere formation assay (g). (h) is the quantification data for (g). **i** PKMYT1AR targeting ASOs inhibited cancer stem cell marker genes expressions examined by immunoblot with indicated antibodies. **j-k** Representative IHC staining of CD44, Sox2 and β-catenin for indicated xenograft tumors (j). (k) is the quantification data for (j). Scale bar=50 μm. * *P* < 0.05, ** *P* < 0.01, *** *P* < 0.001.

**Table S1: The candidate lncRNAs were identified by integrative analyses using various GEO datasets.**

| **GSE81089** | GSE144520 | **GSE157427** |
| --- | --- | --- |
| AL390786.1 | AC092447.4 | ZNF8-ERVK3-1 |
| AC034187.1 | AL441883.1 | AC217774.2 |
| AC009690.2 | AL161629.1 | AC003005.2 |
| AC003005.2 | AP003465.1 | AC107294.1 |
| AC104561.3 | RORA-AS1 | AC079160.1 |
| LRRC7-AS1 | AP005121.1 | AC108868.1 |
| AC078860.2 | AP002856.2 | AC093582.1 |
| AC099788.1 | AC004832.6 | AC016710.1 |
| BX927359.1 | LINC01851 | TSPAN9-IT1 |
| AC025871.2 | LINC01217 | AC138915.3 |
| BET1-AS1 | AC003005.2 | AP000426.1 |
| AC073525.1 | LINC01686 | NEAT1 |
| AC009269.3 | AC099506.1 | FLNC-AS1 |
| AC138207.4 | LINC01091 | AL117190.2 |
| AC073655.1 | NEAT1 | UMODL1-AS1 |
| AP002907.1 | AL354928.1 | LINC02058 |
| LUARIS | LINC02686 | PARD3-AS1 |
| LINC00504 | AC114498.1 | AC103987.2 |
| AC078922.1 | LINC01124 | LINC01124 |
| AC027237.2 | TMEM72-AS1 | AC012574.1 |
| AL590378.1 | EPS15-AS1 | LINC01209 |
| HELLPAR | LINC01531 | AC096734.1 |
| LINC01124 | ZFAND2A-DT | AC087477.2 |
| AC008147.2 | AC080013.3 | AF235103.1 |
| WAC-AS1 |  | AL391863.2 |
| NEAT1 |  | LINC02504 |
| LINC02740 |  | AC015849.3 |
| ITCH-AS1 |  | AL356489.1 |
| AL355300.1 |  | FREM2-AS1 |
| DIRC1 |  | LINC01173 |
| LINC02365 |  |  |
| CREB3L2-AS1 |  |  |
| LINC01792 |  |  |
| LINC01227 |  |  |
| AATBC |  |  |
| AC011374.1 |  |  |
| AC069277.1 |  |  |
| AL049874.3 |  |  |
| DUXAP8 |  |  |
| PRKAG2-AS1 |  |  |
| AL132657.1 |  |  |
| AL109947.1 |  |  |
| AC073127.1 |  |  |
| LINC01679 |  |  |
| AC104063.1 |  |  |
| AC079349.1 |  |  |
| AC012349.1 |  |  |
| AC069133.1 |  |  |
| TMSB15B-AS1 |  |  |
| AL138831.1 |  |  |
| JPX |  |  |
| LINC01340 |  |  |
| AP000786.1 |  |  |
| AL596188.1 |  |  |
| FP325332.1 |  |  |
| LINC01031 |  |  |
| AC138331.1 |  |  |
| AC007879.3 |  |  |

**AC003005.2: PKMYT1AR.**

GSE81089: Next Generation Sequencing (RNAseq) of non-small cell lung cancer. GSE144520: Whole-transcriptome sequencing of A549 cells and cisplatin-resistance A549/DPP cells.

GSE157427: Gene expression profiles for lung cancer cell and lung cancer stem cells.

**Table S2. Antibodies and oligos used in this study.**

| **Antibody Name** | **Catalog number** | **Dilution** | **Supplier** | **Species** |
| --- | --- | --- | --- | --- |
| CDK2 | 10122-1-AP | 1:2000 | Proteintech | Rabbit |
| CDK6 | ab124821 | 1:2000 | abcam | Rabbit |
| Cyclin D1 | 60186-1-1g | 1:1000 | Proteintech | Mouse |
| β-actin | 60008-1-1g | 1:5000 | Proteintech | Mouse |
| p27 | 610241 | 1:2000 | BD | Mouse |
| E-cadherin | ab40772 | 1:500 | abcam | Rabbit |
| N-cadherin | ab18203 | 1:1000 | abcam | Rabbit |
| Vimentin | 103661-1-AP | 1:2000 | Proteintech | Rabbit |
| PARP | 9542S | 1:1000 | CST | Rabbit |
| Cleaved caspase3 | 9661S | 1:500 | CST | Rabbit |
| Bcl-2 | 15071S | 1:500 | CST | Mouse |
| Bax | ab77566 | 1:1000 | abcam | Mouse |
| β-catenin | 610153 | 1:2000 | BD | Mouse |
| PKMYT1 | 200387 | 1:1000 | Abcam | Rabbit |
| Oct4 | ab19857 | 1:1000 | abcam | Rabbit |
| Sox2 | 23064S | 1:1000 | CST | Rabbit |
| HA | sc-7392 | 1:1000 | santa cruz | Mouse |
| Flag | F1804 | 1:1000 | Sigma | Mouse |
| Flag | 14793S | 1:500 | CST | Rabbit |
| CD44 | 3570s | 1:1000 | CST | Mouse |
| **Oligo name** | **Primer sequences (5'-3')** | | | |
| Human β-actin_F | AAGTGTGACGTGGACATCCGC | | | |
| Human β-actin_R | CCGGACTCGTCATACTCCTGCT | | | |
| Human Sox2_F | CACAGATGCAACCGATGCA | | | |
| Human Sox2_R | GGTGCCCTGCTGCGAGTA | | | |
| Human CD44_F | CTGCCGCTTTGCAGGTGTA | | | |
| Human CD44_R | CATTGTGGGCAAGGTGCTATT | | | |
| Human Oct4_F | CTGGGTTGATCCTCGGACCT | | | |
| Human Oct4_R | CCATCGGAGTTGCTCTCCA | | | |
| Human Nanog_F | TTTGTGGGCCTGAAGAAAACT | | | |
| Human Nanog_R | AGGGCTGTCCTGAATAAGCAG | | | |
| Human PKMYT1_F | CATGGCTCCTACGGAGAGGT | | | |
| Human PKMYT1_R | ACATGGAACGCTTTACCGCAT | | | |
| Human Axin2_F | AGTCAGCAGAGGGACAGGAA | | | |
| Human Axin2_R | CTTCGTACATGGGGAGCACT | | | |
| Human c-Myc_F | GGCTCCTGGCAAAAGGTCA | | | |
| Human c-Myc_R | CTGCGTAGTTGTGCTGATGT | | | |
| Human CyclinD1_F | GCTCCTGTGCTGCGAAGT | | | |
| Human CyclinD1_R | TGTTCCTCTCAGACCTCCAG | | | |
| Human YY1_F | ACGGCTTCGAGGATCAGATTC | | | |
| Human YY1_R | TGACCAGCGTTTGTTCAATGT | | | |
| PKMYT1AR_F | CCACGGCACCAACACTAGTA | | | |
| PKMYT1AR_R | ATCTCAGCACTTTGGGAGGC | | | |
| Human PKMYT1AR sh#1 Forward oligos | CCGGGCTTAGCTTCCTCTTGAAGGACTCGAGTCCTTCAAGAGGAAGCTAAGCTTTTTG | | | |
| Human PKMYT1AR sh#1 Reverse oligos | AATTCAAAAAGCTTAGCTTCCTCTTGAAGGACTCGAGTCCTTCAAGAGGAAGCTAAGC | | | |
| Human PKMYT1AR sh#2 Forward oligos | CCGGGCCTTCCTTACGCAGAGAATGCTCGAGCATTCTCTGCGTAAGGAAGGCTTTTTG | | | |
| Human PKMYT1AR sh#2 Reverse oligos | AATTCAAAAAGCCTTCCTTACGCAGAGAATGCTCGAGCATTCTCTGCGTAAGGAAGGC | | | |
| Human PKMYT1 sh#1 Forward oligos | CCGGGCAGCGGATGTGTTCAGTCTGCTCGAGCAGACTGAACACATCCGCTGCTTTTTG | | | |
| Human PKMYT1 sh#1 Reverse oligos | AATTCAAAAAGCAGCGGATGTGTTCAGTCTGCTCGAGCAGACTGAACACATCCGCTGC | | | |
| Human PKMYT1 sh#2 Forward oligos | CCGGGCACTGCCTGTGTTGAGGCAGCTCGAGCTGCCTCAACACAGGCAGTGCTTTTTG | | | |
| Human PKMYT1 sh#2 Reverse oligos | AATTCAAAAAGCACTGCCTGTGTTGAGGCAGCTCGAGCTGCCTCAACACAGGCAGTGC | | | |
| Human YY1 sh#1 Forward oligos | CCGGGAACTCACCTCCTGATTATTCCTCGAGGAATAATCAGGAGGTGAGTTCTTTTTG | | | |
| Human YY1 sh#1 Reverse oligos | AATTCAAAAAGAACTCACCTCCTGATTATTCCTCGAGGAATAATCAGGAGGTGAGTTC | | | |
| Human YY1 sh#2 Forward oligos | CCGGGAAGATGATGCTCCAAGAACACTCGAGTGTTCTTGGAGCATCATCTTCTTTTTG | | | |
| Human YY1 sh#2 Reverse oligos | AATTCAAAAAGAAGATGATGCTCCAAGAACACTCGAGTGTTCTTGGAGCATCATCTTC | | | |
| Hsa-miR-485-p_qPCR | CCAAGCTTCACCCATTCCTAACAGGAC | | | |
| Human U6_qPCR | CTCGCTTCGGCAGCACA | | | |
| PKMYT1AR ASO#1 | GGCCTTGAAGCTGGAGTGCA | | | |
| PKMYT1AR ASO#2 | GCCATATCTGTATTTCTGGT | | | |

**Table S3: Top 50% predicted downstream target genes of miRNA-485-5p examined by StarBase, Targetscan and miRWalk, respectively.**

| **StarBase** | **Targetscan** | **miRWalk** |
| --- | --- | --- |
| UBE2J2 | PTMS | PAX3 |
| DVL1 | AC007390.5 | ANXA11 |
| MXRA8 | SUMF2 | AGFG2 |
| MRPL20 | DNAJC5G | FAM83F |
| SLC35E2B | UNC45A | DAG1 |
| GNB1 | SSH3 | FBXL17 |
| TMEM52 | RAB8B | AGO4 |
| LRRC47 | ZBTB39 | DNAJB13 |
| RPL22 | CKS1B | POU6F1 |
| NOL9 | TPD52L2 | SOX5 |
| KLHL21 | HIF3A | ZNF831 |
| DNAJC11 | MEOX1 | PEX26 |
| ENO1 | ALX4 | NKAIN4 |
| GPR157 | PPP1R14D | RASL10A |
| DFFA | CES4A | TRIM8 |
| **PKMYT1** | ONECUT2 | MSI2 |
| MTOR | TMEM151B | OSBPL10 |
| MAD2L2 | SLC36A3 | SGPL1 |
| MTHFR | C7orf73 | CD79A |
| DHRS3 | CD3EAP | RRM2 |
| HSPB7 | PHB2 | VPS36 |
| RCC2 | **PKMYT1** | ORAI1 |
| EMC1 | DLK2 | **PKMYT1** |
| CAPZB | TMUB2 | BTG2 |
| HP1BP3 | PAK1 | SLC36A1 |
| ECE1 | SDC3 | CHD3 |
| HSPG2 | TSPO2 | TAB1 |
| LUZP1 | TFRC | S100A16 |
| E2F2 | SRRM4 | IGF2BP2 |
| RUNX3 | YWHAB | ATG9B |
| NR0B2 | C17orf50 | FUNDC2 |
| SLC9A1 | ZNF384 | LYPD6 |
| WASF2 | FAM124A | SGSM2 |
| IFI6 | VTA1 | CD84 |
| TAF12 | RMND5B | MBD6 |
| SRSF4 | KNOP1 | DRGX |
| PUM1 | ETNK2 | GPN2 |
| NKAIN1 | TMEM104 | ZSWIM6 |
| ZBTB8OS | AJAP1 | ZNF259 |
| TRIM62 | ZNF81 | PRUNE |
| PHC2 | ADIPOR2 | KCNK3 |
| STK40 | NSUN5 | EMC10 |
| CSF3R | HOXB6 | SNX21 |
|  | KPRP | DISP2 |
|  | ACTR3 | ARHGAP9 |
|  | RNF24 | TEX261 |
|  | VPS26A | C2orf91 |
|  | ATOH8 | KNTC1 |
|  | LSMEM2 | ZCCHC14 |
|  | FAM222B | HNF4A |
|  | EFNA1 | UBA2 |
|  | MRPS27 | LZTS3 |
|  | TIMM13 | PANK3 |
|  | FRMPD3 | GPR3 |
|  | NNT | B3GNT1 |
|  | EVX2 | FOXL1 |
|  | ASCC2 | ANKRD52 |
|  | NOX5 | UBR2 |
|  | NT5DC3 | UPF2 |
|  | FSD1 | KLF13 |
|  | MPZ | RWDD2B |
|  | CTDNEP1 | KCNJ11 |
|  | GGT7 | YWHAG |
|  | TDRKH | BAZ2A |
|  | PARP11 | CAMK2A |
|  | MAX | ST14 |
|  | TOM1L2 | MCTP1 |
|  | ARL2BP | CLCN6 |
|  | PAPLN | C9orf114 |
|  | DAND5 | NMNAT2 |
|  | DCAF7 | FKBP4 |
|  | PPAN-P2RY11 | CTD-2207O23.12 |
|  | AVPR2 | SEMA4G |
|  | SPNS2 | ZNF641 |
|  | MGAT5B | GJA5 |
|  | SLC16A2 | LCMT2 |
|  | NF2 | FRMD5 |
|  | FTCD | CEP85 |
|  | LRRC27 | TP53INP2 |
|  | CYB561D1 | COPS7B |
|  | ZFP36L1 | LYSMD1 |
|  | RBPMS | H6PD |
|  | IL18BP | AC022532.1 |
|  | PDPR | SYT11 |
|  | CDC42EP1 | RP5-850E9.3 |
|  | DKFZP434H0512 | MIER2 |
|  | PEAR1 | PAK4 |
|  | PACS1 | SLC25A53 |
|  | DKFZP761J1410 | RORC |
|  | MGST3 | CDKN1A |
|  | C17orf78 | ANKRD34A |
|  | ST3GAL1 | TSTD2 |
|  | GAB2 | ZBTB20 |
|  | BCAS3 | MXI1 |
|  | DHCR24 | FXYD5 |
|  | CSNK2A1 | BEND6 |
|  | GSK3A | POMGNT1 |
|  | MAP7D1 | LAMTOR1 |
|  | CALCOCO1 | GABRE |
|  | EPS8L1 | TMEM181 |
|  | CDX1 | TTC9C |
|  | FAM83B | LCLAT1 |
|  |  | APOA5 |
|  |  | SLC41A1 |
|  |  | PQLC2 |
|  |  | TAOK1 |
|  |  | PFN2 |
|  |  | ELMSAN1 |
|  |  | DPYSL3 |
|  |  | TSPAN17 |
|  |  | IL23R |
|  |  | ZDHHC2 |
|  |  | CCAR2 |
|  |  | CELF3 |
|  |  | ABHD2 |
|  |  | MXD1 |
|  |  | JUND |
|  |  | NPLOC4 |
|  |  | PPP4R1L |
|  |  | GINS4 |
|  |  | ZDHHC8 |
|  |  | MEF2D |
|  |  | SF1 |
|  |  | DMWD |
|  |  | NPEPPS |
|  |  | DNM1 |
|  |  | SLC2A3 |

**Table S4: The full names of cancer types shown in Fig. S4a.**

| **cancer type** | **Full name** |
| --- | --- |
| ACC | Adrenocortical carcinoma |
| BLCA | Bladder Urothelial Carcinoma |
| BRCA | Breast invasive carcinoma |
| CESC | Cervical squamous cell carcinoma and endocervical adenocarcinoma |
| CHOL | Cholangiocarcinoma |
| COAD | Colon adenocarcinoma |
| DLBC | Lymphoid Neoplasm Diffuse Large B-cell Lymphoma |
| ESCA | Esophageal carcinoma |
| GBM | Glioblastoma multiforme |
| HNSC | Head and Neck squamous cell carcinoma |
| KICH | Kidney Chromophobe |
| KIRC | Kidney renal clear cell carcinoma |
| KIRP | Kidney renal papillary cell carcinoma |
| LAML | Acute Myeloid Leukemia |
| LGG | Brain Lower Grade Glioma |
| LIHC | Liver hepatocellular carcinoma |
| LUAD | Lung adenocarcinoma |
| LUSC | Lung squamous cell carcinoma |
| MESO | Mesothelioma |
| OV | Ovarian serous cystadenocarcinoma |
| PAAD | Pancreatic adenocarcinoma |
| PCPG | Pheochromocytoma and Paraganglioma |
| PRAD | Prostate adenocarcinoma |
| READ | Rectum adenocarcinoma |
| SARC | Sarcoma |
| SKCM | Skin Cutaneous Melanoma |
| STAD | Stomach adenocarcinoma |
| TGCT | Testicular Germ Cell Tumors |
| THCA | Thyroid carcinoma |
| THYM | Thymoma |
| UCEC | Uterine Corpus Endometrial Carcinoma |
| UCS | Uterine Carcinosarcoma |
| UVM | Uveal Melanoma |

**Table S5: The dataset used in the Fig. S4c.**

| Cancer type | Cancerstudy | DOI | Journal |
| --- | --- | --- | --- |
| #1 | Diffuse Large B-Cell Lymphoma (TCGA, PanCancer Atlas) | [DOI: 10.1016/j.cell.2018.03.022](https://doi.org/10.1016/j.cell.2018.03.022) | Cell |
| #2 | Breast Invasive Carcinoma (TCGA, PanCancer Atlas) | [DOI: 10.1016/j.cell.2018.03.022](https://doi.org/10.1016/j.cell.2018.03.022) | Cell |
| #3 | Uterine Corpus Endometrial Carcinoma (TCGA, PanCancer Atlas) | [DOI: 10.1016/j.ccell.2018.03.007](https://doi.org/10.1016/j.ccell.2018.03.007) | Cancer Cell |
| #4 | Cholangiocarcinoma (TCGA, PanCancer Atlas) | [DOI: 10.1016/j.cell.2018.02.052](https://doi.org/10.1016/j.cell.2018.02.052) | Cell |
| #5 | Stomach Adenocarcinoma (TCGA, PanCancer Atlas) | [DOI: 10.1016/j.cell.2018.03.033](https://doi.org/10.1016/j.cell.2018.03.033) | Cell |
| #6 | Esophageal Adenocarcinoma (TCGA, PanCancer Atlas) | [DOI: 10.1016/j.cell.2018.03.033](https://doi.org/10.1016/j.cell.2018.03.033) | Cell |
| #7 | Ovarian Serous Cystadenocarcinoma (TCGA, PanCancer Atlas) | [DOI: 10.1016/j.celrep.2018.03.050](https://doi.org/10.1016/j.celrep.2018.03.050) | Cell Rep |
| #8 | Bladder Urothelial Carcinoma (TCGA, PanCancer Atlas) | [DOI: 10.1016/j.cell.2018.03.035](https://doi.org/10.1016/j.cell.2018.03.035) | Cell |
| #9 | Skin Cutaneous Melanoma (TCGA, PanCancer Atlas) | [DOI: 10.1200/PO.17.00073](https://doi.org/10.1200/po.17.00073) | JCO Precis Oncol |
| #10 | Colorectal Adenocarcinoma (TCGA, PanCancer Atlas) | [DOI: 10.1038/s41586-020-2095-1](https://doi.org/10.1038/s41586-020-2095-1) | Nature |
| #11 | Prostate Adenocarcinoma (TCGA, PanCancer Atlas) | [DOI: 10.1038/s41588-018-0318-2](https://doi.org/10.1038/s41588-018-0318-2) | Nat Genet |
| #12 | Cervical Squamous Cell Carcinoma (TCGA, PanCancer Atlas) | [DOI: 10.1016/j.cels.2018.03.002](https://doi.org/10.1016/j.cels.2018.03.002) | Cell Syst |
| #13 | Sarcoma (TCGA, PanCancer Atlas) | [DOI: 10.1016/j.cell.2018.02.052](https://doi.org/10.1016/j.cell.2018.02.052) | Cell |
| #14 | Adrenocortical Carcinoma (TCGA, PanCancer Atlas) | [DOI: 10.1016/j.cell.2018.02.052](https://doi.org/10.1016/j.cell.2018.02.052) | Cell |
| #15 | Lung Adenocarcinoma (TCGA, PanCancer Atlas) | [DOI: 10.1038/nature13385](https://doi.org/10.1038/nature13385) | Nature |
| #16 | Lung Squamous Cell Carcinoma (TCGA, PanCancer Atlas) | [DOI: 10.1038/nature11404](https://doi.org/10.1038/nature11404) | Nature |
| #17 | Kidney Renal Papillary Cell Carcinoma (TCGA, PanCancer Atlas) | [DOI: 10.1038/ng.2699](https://doi.org/10.1038/ng.2699) | Nat Genet |
| #18 | Testicular Germ Cell Tumors (TCGA, PanCancer Atlas) | [DOI: 10.1158/1078-0432.CCR-15-2867-T](https://doi.org/10.1158/1078-0432.ccr-15-2867-t) | Clin Cancer Res |
| #19 | Pancreatic Adenocarcinoma (TCGA, PanCancer Atlas) | [DOI: 10.1038/nature12222](https://doi.org/10.1038/nature12222) | Nature |
| #20 | Liver Hepatocellular Carcinoma (TCGA, PanCancer Atlas) | [DOI: 10.1371/journal.pone.0200776](https://doi.org/10.1371/journal.pone.0200776) | PLoS One |
| #21 | Glioblastoma Multiforme (TCGA, PanCancer Atlas) | [DOI: 10.1016/j.cell.2018.03.022](https://doi.org/10.1016/j.cell.2018.03.022) | Cell |
| #22 | Brain Lower Grade Glioma (TCGA, PanCancer Atlas) | [DOI: 10.1038/s41591-019-0349-y](https://doi.org/10.1038/s41591-019-0349-y) | Nat Med |
| #23 | Head and Neck Squamous Cell Carcinoma (TCGA, PanCancer Atlas) | [DOI: 10.1016/j.cell.2018.03.022](https://doi.org/10.1016/j.cell.2018.03.022) | Cell |
| #24 | Thyroid Carcinoma (TCGA, PanCancer Atlas) | [DOI: 10.1158/2159-8290.CD-13-0617](https://doi.org/10.1158/2159-8290.cd-13-0617) | Cancer Discov |

**Table S6. The pathological characteristics of patients with non-small cell lung cancer (NSCLC), and health donors.**

| Patients characteristics | No. (%) |
| --- | --- |
| **NSCLC patients** |  |
| **Age(years)** |  |
| ≤50 | 100(44.8%) |
| ＞50 | 123(55.2%) |
| **Gender** |  |
| Male | 109(48.8%) |
| Female | 114(51.2%) |
| **Health donor** |  |
| **Age(years)** |  |
| ≤50 | 34(46.5%) |
| ＞50 | 39(53.5%) |
| **Gender** |  |
| Male | 40(54.7%) |
| Female | 33(45.3 %) |

**Ethics Statement**

Samples were obtained with informed consent and all protocols were approved by The Second Xiangya Hospital of Central South University Ethics Review Board (Scientific and Research Ethics Committee, S-02/2000). Written informed consent was obtained from all patients，also the written informed consent was obtained from the next of kin, caretakers, or guardians on the behalf of the minors/children participants involved in your study.
